# Lung Ultrasound in the Evaluation of Lung Disease Severity in Children with Clinically Stable Cystic Fibrosis: A Prospective Cross-Sectional Study

**DOI:** 10.3390/jcm12093086

**Published:** 2023-04-24

**Authors:** Joanna Jaworska, Natalia Buda, Piotr Kwaśniewicz, Anna Komorowska-Piotrowska, Dorota Sands

**Affiliations:** 1 Cystic Fibrosis Department, Institute of Mother and Child, 01-211 Warsaw, Poland; dorota.sands@imid.med.pl; 2 Department of Internal Medicine, Connective Tissue Diseases and Geriatrics, Medical University of Gdansk, 80-214 Gdansk, Poland; natabud83@gmail.com; 3 Department of Diagnostic Imaging, Institute of Mother and Child, 01-211 Warsaw, Poland; kwasniewiczp@gmail.com; 4 Specialist Centre for Diagnostics and Therapy of a Small Child BALUMED, 02-972 Warsaw, Poland; komorowskapiotrowska.anna@gmail.com

**Keywords:** lung ultrasound, LUS, cystic fibrosis, CF, CF LUS score, Am-lines, interobserver agreement

## Abstract

With the increasing longevity of cystic fibrosis (CF), there is a growing need to minimise exposure to ionising radiation in patients who undergo regular imaging tests while monitoring the course of the lung disease. This study aimed to define the role of lung ultrasounds (LUS) in the evaluation of lung disease severity in children with clinically stable CF. LUS was performed on 131 patients aged 5 weeks to 18 years (study group) and in 32 healthy children of an equivalent age range (control group). Additionally, an interobserver study was performed on 38 patients from the study group. In CF patients, the following ultrasound signs were identified: I-lines; Z-lines; single, numerous and confluent B-lines; Am-lines; small and major consolidations; pleural line abnormalities and small amounts of pleural fluid. The obtained results were evaluated against an original ultrasound score. LUS results were correlated with the results of chest X-ray (CXR) [very high], pulmonary function tests (PFTs) [high] and microbiological status [significant]. The interobserver study showed very good agreement between investigators. We conclude that LUS is a useful test in the evaluation of CF lung disease severity compared to routinely used methods. With appropriate standardisation, LUS is highly reproducible.

## 1. Introduction

Cystic fibrosis (CF) is one of the most common autosomal recessive genetic diseases. Its course has changed dramatically over the last few decades—from a lethal disease of early childhood, it has become a chronic disease with a life expectancy of around 50 years for currently born children [1,2]. Progressive lung disease is a leading manifestation of this multisystem disorder. Adequate CF management requires monitoring the disease’s course and response to the applied therapy. Hence, periodic assessments of patients with clinically stable CF constitute a crucial element of their medical care. Essential tools for evaluating lung disease severity include lung function measurements, cultures of respiratory specimens and imaging studies.

Despite its limitations, chest X-ray (CXR) remain the first-choice imaging modality for annual clinical assessments in most paediatric CF centres [3,4]. In comparison to CXR, chest computed tomography (chest CT) is much more sensitive [5,6,7,8] but exposes patients to substantially higher ionising radiation doses, even with modern low-dose protocols [9,10,11]. With the longer lifespans of CF patients, the cumulative radiation dose and carcinogenic risk increase [12], prompting clinicians to search for safer solutions, especially in the paediatric population. Magnetic resonance (MRI) is an excellent [13,14,15] but demanding alternative. It is an expensive, time-consuming procedure which requires lengthy training time and either sedation or general anaesthetic in younger children. Consequently, lung ultrasonography (LUS) appears to be a promising method. It is a radiation-free, inexpensive, widely available procedure with a relatively short performing time and an encouraging learning curve, available at the bedside and is less dependent on the child’s movement (including crying) [16,17].

At the planning phase of this study (2015), there were no English-language publications on the use of LUS in CF, except for two short conference reports [18,19]. The same authors presented two more conference reports in 2018 [20,21]. Subsequently, five articles assessing the diagnostic value of LUS in CF have been published [22,23,24,25,26]. Since these studies were conducted simultaneously with the presented work, comparison will be included in the discussion.

## 2. Materials and Methods

### 2.1. Study, Control and Interobserver Group

The study was conducted on 131 patients with clinically stable CF (58 girls—44% and 73 boys—56%) aged five weeks to eighteen years (median 7.1 years) during scheduled follow-up visits at the Warsaw Cystic Fibrosis Centre between May 2016 and December 2019. In a great majority of cases, the CF diagnosis had been established through new-born screening, confirmed in almost all children by two positive sweat test results and—in all cases—by genetic test results (2 pathogenic mutations of the *CFTR* gene). Exclusion criteria were as follows: lack of consent to participate in the study, pulmonary exacerbation (PEx), cough exaggeration or other clinical symptoms of acute respiratory tract infection during the 6 weeks preceding the examination, suspicion or diagnosis of acute CF complications (hemoptysis, pneumothorax, distal intestinal obstruction syndrome) and other emergencies (e.g., acute pancreatitis).

The control group consisted of 32 healthy children (18 girls—56% and 14 boys—44%) aged two months to eighteen years (median 6.7 years). Exclusion criteria were as follows: lack of consent to participate in the study, CF or another chronic respiratory disease, cough or other clinical symptoms of acute respiratory tract infection during the 6 weeks preceding the examination.

The interobserver group comprised 38 children from the study group (13 girls—34% and 25 boys—66%) aged five weeks to sixteen and a half years (median 8.3 years).

### 2.2. Study Design

All enrolled children underwent LUS performed by one sonographer, a paediatric pulmonologist who, at the beginning of the study, had over one year of sonographic experience in the field. Additionally, the interobserver group was also examined by a second sonographer (a paediatric radiologist with over 7 years’ experience) within 24 h of the first LUS. Both researchers were blinded regarding patients’ previous workup. In the study group, the remaining diagnostic tests were carried out within 72 h of LUS. CXR was performed on 83 patients, spirometry on 77 patients, and multiple breath nitrogen washout (MBNW) on 64 patients. In all 131 children, microbiological status was assessed.

### 2.3. Diagnostic Methods

**LUS** was performed with the use of Xario 100 (Toshiba, Tokyo Japan, TUS X-100), with two probes—convex (3.3–9.2 MHz) and linear (5.0–18.0 MHz). The preliminary preset was abdominal (for convex) or soft tissue (for linear transducer) with the exclusion of artefact reduction modes (e.g., SonoCT, XRes). Colour Doppler imaging was utilised for lesion vascularisation assessment. The mechanical index was set to between 0.4 and 0.6 for safety reasons [17,27,28,29]. In most children, the anterior chest was examined in the supine position, and the posterior chest and the lung apices were examined in the sitting position (the borders between anterior and posterior surfaces were the midaxillary lines). Infants were examined in the supine and prone positions. Exceptions to this rule were tests performed on children who, due to anxiety, needed to stay on their caregivers’ laps. The entire available lung surface was evaluated—each intercostal space and both supraclavicular areas. The transducers were placed in the intercostal spaces, transverse to the axis of the body and moved continuously from the apex to the base of the lung. In case of any findings, the probes were also placed longitudinally. The presence of ultrasound (US) signs was determined for 12 lung fields—6 anterior (upper, middle and lower; both on the right and the left sides) and 6 posterior (Figure S1) [30]. The obtained LUS results were evaluated according to the ultrasound score designed for the purpose of this study (CF LUS score) for four lung areas (grouped three lung fields each: 1. left anterior; 2. right anterior; 3. left posterior; 4. right posterior) (Table 1).

Before the interobserver study was conducted, the two sonographers discussed and standardised the examination method and the LUS findings’ definitions. Interobserver agreement was assessed for individual US signs and the results of the CF LUS score.

**Figure 1:**
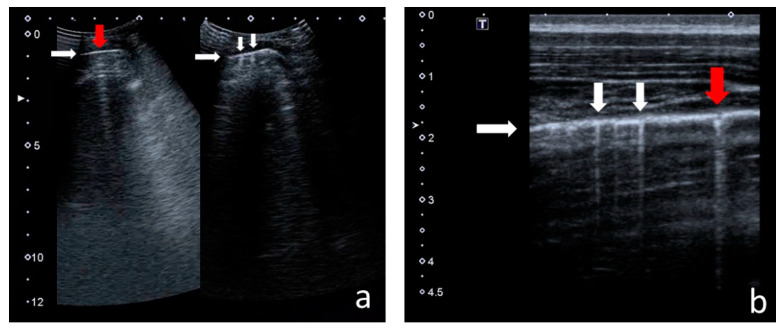
I-lines and Z-lines in (**a**) convex probe; (**b**) linear probe. White horizontal arrows—pleural line, white vertical arrows—I-lines, red arrows—Z-lines.

**Figure 2:**
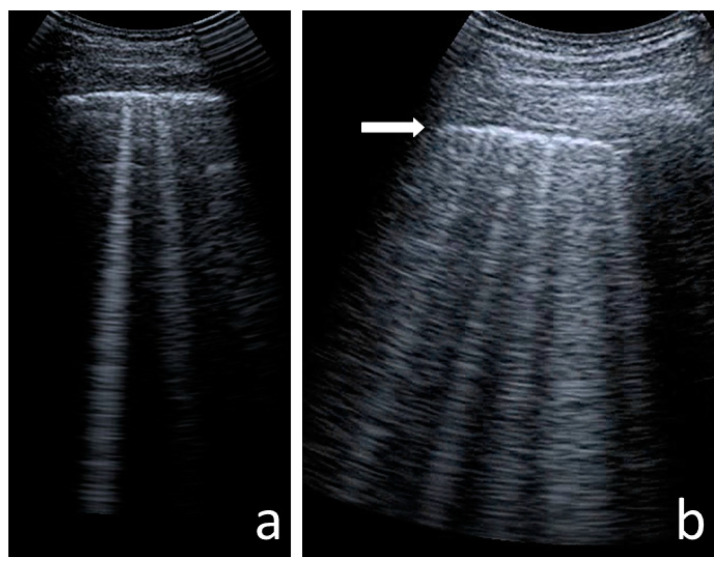
B-lines (**a**) single; (**b**) numerous. White arrow—irregular pleural line.

**Figure 3:**
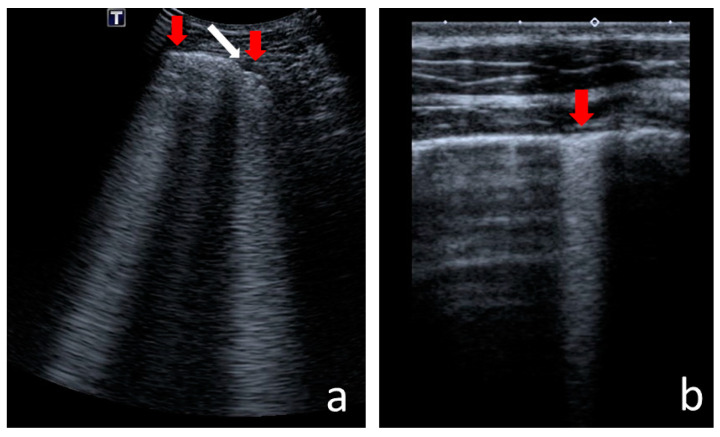
Confluent B-lines in (**a**) convex probe; (**b**) linear probe. Red arrows—confluent B-lines, white arrow—ragged pleural line.

**Figure 4:**
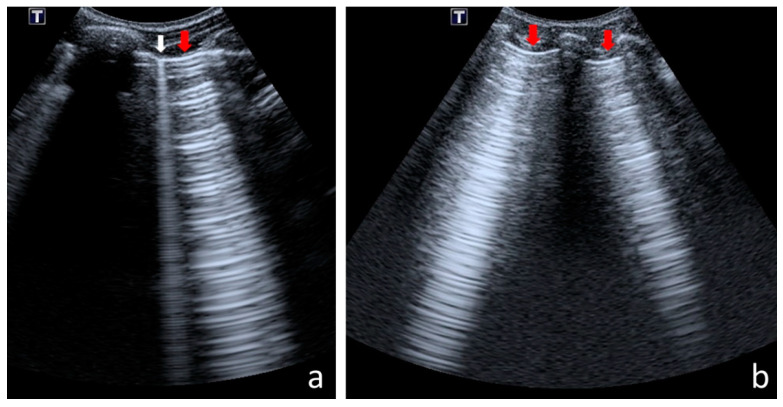
Am-lines (**a**) next to B-line; (**b**) in adjacent intercostal spaces. Red arrows—Am-lines, white arrow—B-line.

**Figure 5:**
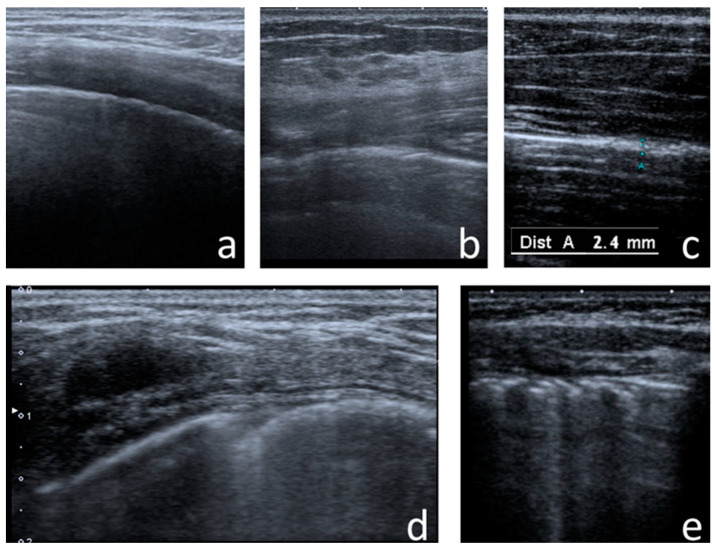
Abnormal pleural line: (**a**) irregular; (**b**) blurred; (**c**) thickened; (**d**) ragged (some sonographers would describe a small consolidation in this case); (**e**) fragmentary.

**Figure 6:**
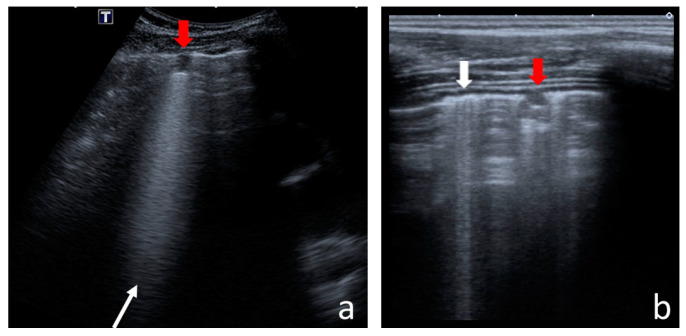
Small consolidations in (**a**) convex probe; (**b**) linear probe. Red arrows—consolidations, vertical white arrow—B-line, oblique white arrow—C-line.

**Figure 7:**
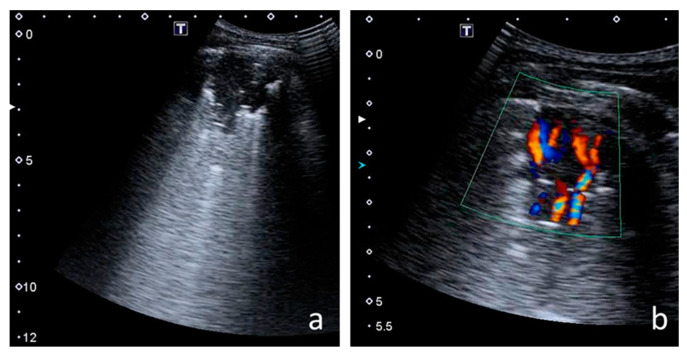
Major consolidation: (**a**) with scanty, static air bronchogram/air trapping and C-lines arising from the irregular deep edge of the consolidation; (**b**) with a tree-like (anatomical) vascular pattern, but densely spaced—atelectatic lesion.

The following US signs were visualised:

- A-lines—hyperechoic, horizontal artefacts that appear at regular intervals, equalling the distance between the body surface and the pleural line [17,31];
- I-lines—hyperechoic, short (1–2 cm), vertical artefacts arising from the pleural line, moving in concert with lung sliding [32] (Figure 1);
- Z-lines—hyperechoic, relatively short (1/3–1/2 of the screen), vertical artefacts arising from the pleural line [31,32,33] (Figure 1);
- B-lines—hyperechoic, long (usually ending at the screen’s lower edge) vertical artefacts (also called “comet-tail artefacts”) arising from the pleural line, moving in concert with lung sliding, usually “erasing” A-lines [17,31] (Figure 2 and Figure 3);
- Am-lines—broad vertical artefacts (narrower at the top and broader at the bottom) consisting of multiple horizontal artefacts arranged parallelly, ending at the screen’s lower edge [34] (Figure 4);
- pleural line abnormalities: irregularity, fragmentation, blurring, ragging and thickening [17] (Figure 5);
- C-lines—hyperechoic, vertical artefacts arising from the deep edge of a consolidation; in major consolidations, the deep edge is usually irregular (shred sign) [17,31] (Figure 6 and Figure 7);
- Consolidations—hypoechoic, tissue-like areas: (a) small (≤10 mm) (Figure 6), and (b) major (>10 mm) (Figure 7); usually, major consolidations have the following associated features: the loss of pleural line echogenicity over the area of consolidation, absence of A-lines, presence of dynamic or static air bronchogram/air trapping and vascular pattern (in CD option) within the area, C-lines below the area, B-lines surrounding it [17,31];
- pleural fluid [17,31].

**CXR** was performed according to the routine schedule of annual check-ups: in younger children (infants and younger toddlers) using the anteroposterior view in the supine position, and in older children using the posteroanterior view in the standing position. Radiology examinations were executed with digital devices: Axiom Iconos R200, Apollo Villa (Siemens), Perform-X Radiographic Phoenix System (Control-X Medical) and Luminos dRF MAX3D (Siemens). The technical parameters (grid, source to image-receptor distance, irradiation dose) were adjusted individually according to the ALARA (as low as reasonably achievable) principle. A paediatric radiologist with experience in CF evaluated the images against the modified Chrispin–Norman score (a score used to provide a summative assessment of structural lung changes in patients with CF on the frontal chest radiograph) (Table S1) [35,36].

**Pulmonary function tests (PFTs)** were performed in accordance with the European Respiratory Society/American Thoracic Society criteria [37,38,39,40]. The Jaeger Vyntus IOS (CareFusion, Hochberg, Germany) was used to conduct spirometry, and Exhalyzer-D (EcoMedics AG, Duernten, Switzerland, software version 3.2.0) was used for MBNW. All instruments were calibrated on the day of the examination. CF patients underwent PFTs after completing their standard daily airway clearance therapy [41]. The following parameters were considered for the analysis: forced expiratory volume in 1 s (FEV1) and forced vital capacity (FVC) shown as percentage of the predicted value, and lung clearance index (LCI) 2.5%.

**The microbiological status** of the patients was evaluated by sputum culture or throat-swab culture (for children who did not expectorate sputum). The presence of so-called CF pathogens was considered a risk factor of advanced CF lung diseases. (These classical and emerging bacterial pathogens are capable of causing lung damage in the course of CF [42,43,44].) The study group was analysed according to two categories: patients with and without CF pathogens. The history of previous cultures was also reviewed to determine bacteriological status comprehensively. Based on their microbiological status, patients were divided into four subgroups: (1) first time, (2) previously reported, (3) intermittent, and (4) chronic infection. Fungal culture was also performed in children who expectorated sputum, and the history concerning previous fungal infections was analysed. There were no nontuberculous mycobacteria (NTM) infections reported in patients from the study group.

### 2.4. Statistical Analysis

Statistical analysis was performed in R environment, version 4.1.2, under the GNU General Public License. Additionally, strings and epiR packages were used.

Basic sample statistics were determined for quantitative data: mean with standard deviation, quartiles, the lowest and the highest values. The distributions of quantitative variables were presented in histograms or box plots. In the case of qualitative variables, individual values (categories) were counted and presented as numbers, percentages, and bar charts. Percentage estimates for qualitative traits were supplemented with 95% confidence intervals (CI). Based on the available data, using additional calculations, many new variables were created (for example, the number of lung fields with the presence of a given ultrasound sign). In the case of comparing the means of two groups, the non-parametric Mann–Whitney–Wilcoxon (or Wilcoxon rank-sum) test was used; if the distribution of the examined feature was not normal, then the student’s *t*-test was used for independent samples. In the case of comparing the means of more than two groups, the Kruskal–Wallis test was applied. The proportions in the groups were compared using the test of equal proportions. *p* < 0.05 was accepted as the level of significance.

For pairs of selected quantitative variables, Pearson’s correlation coefficient was estimated, and the existence of a relationship between these variables was tested based on this coefficient. Additionally, the data were visualised on scatter plots. In the case of a clear linear relationship, the regression line equation was determined using the least squares method with the value of the determination coefficient R2. Fisher’s exact test was used to examine the dependencies of qualitative traits (2 × 2 contingency tables).

The kappa coefficient in Altman’s interpretation [45], corrected for incidence and bias [46], was used to test the interobserver agreement in the case of zero-one data (Table S2). The concordance correlation coefficient [47] and/or the estimation of the slope of simple linear regression for quantitative data were used to test the interobserver agreement. Guilford’s classification (Table S3) was used to determine the magnitude of correlation [48].

### 2.5. Ethical Aspects

The study was approved by the Bioethics Committee of the Institute of Mother and Child in Warsaw, Poland—Opinion No. 34/2015, dated 09.12.2015. Written informed consent was obtained from each participant’s parent or legal guardian and from every child who was capable of signing (obligatory for children aged 16 and above—according to Polish law).

## 3. Results

### 3.1. US Signs Found in CF Patients vs. Healthy Children

In CF patients, the following ultrasound signs were identified: I-lines; Z-lines; single, numerous and confluent B-lines; Am-lines; small and major consolidations; pleural line abnormalities and small amounts of pleural fluid.

#### 3.1.1. Artefacts

##### I-Line and Z-Line Artefacts

**Figure 8:**
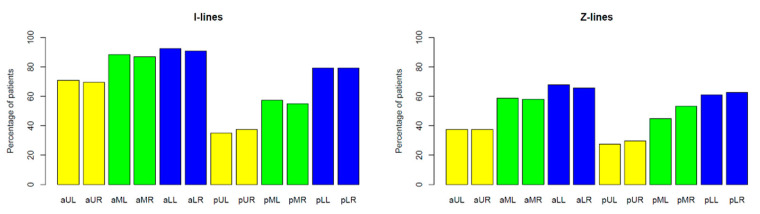
Incidence of I-lines and Z-lines in individual lung fields. Lung fields: **aUL**—anterior upper left, **aUR**—anterior upper right, **aML**—anterior middle left, **aMR**—anterior middle right, **aLL**—anterior lower left, **aLR**—anterior lower right, **pUL**—posterior upper left, **pUR**—posterior upper right, **pML**—posterior middle left, **pMR**—posterior middle right, **pLL**—posterior lower left, **pLR**—posterior lower right.

In CF patients, I-lines and Z-lines (Figure 1) occurred more frequently over a larger area of the lungs and were bigger in number than in healthy children. I-lines were found in 123 patients (94% of the study group) and 17 healthy children (53% of the control group) in at least one lung field. Z-lines were found in 108 patients (82%) and 15 healthy children (47%) in at least one lung field (Table 2). On average, CF patients presented with Z-lines occurring in six lung fields. Z-lines were present in all 12 lung fields in 23% of the study group. In healthy children, single Z-lines were found in single (one to three) lung fields. The incidence of I-lines and Z-lines in individual lung fields in the study group is shown in Figure 8.

##### B-Line Artefacts

**Figure 9:**
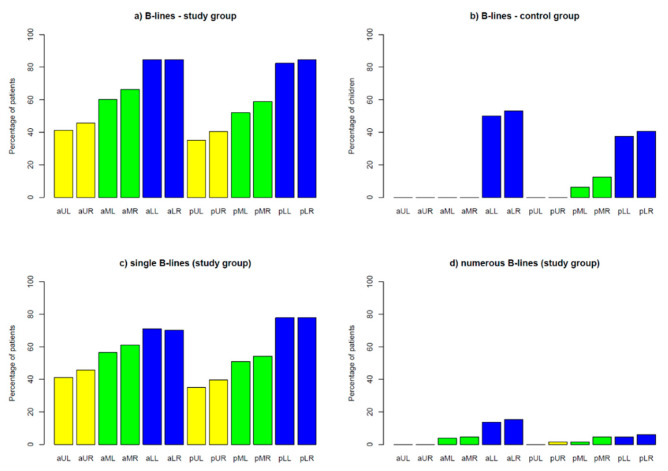
Incidence of B-lines in individual lung fields (**a**) in the study group; (**b**) in the control group; (**c**) single B-lines in the study group; (**d**) numerous B-lines in the study group. Lung fields: **aUL**—anterior upper left, **aUR**—anterior upper right, **aML**—anterior middle left, **aMR**—anterior middle right, **aLL**—anterior lower left, **aLR**—anterior lower right, **pUL**—posterior upper left, **pUR**—posterior upper right, **pML**—posterior middle left, **pMR**—posterior middle right, **pLL**—posterior lower left, **pLR**—posterior lower right.

A total of 99% of children in the study group and only 72% of children in the control group had at least one B-line (Table 2). In CF patients, single B-lines (≤3 per scan) (Figure 2a) were visualised in all lung fields (Figure 9a). Meanwhile, in healthy children, single B-lines did not occur in the upper lung fields and occurred only rarely (19%) in the middle lung fields (Figure 9b). On average, CF patients presented with single B-lines occurring in six lung fields. Single B-lines were present in at least nine lung fields in 25% of the study group. Numerous B-lines (≥4 per scan) (Figure 2b) and confluent (coalescent) B-lines (Figure 3) were found exclusively in the study group—in 27% and 5% of patients, respectively (Table 2). Of the children in the study group, 70% had only single B-lines. In 29% of CF patients, not only single but also numerous and/or confluent B-lines were found. Numerous B-lines were described mainly in the lower lung fields (Figure 9d).

##### Am-Line Artefacts

**Figure 10:**
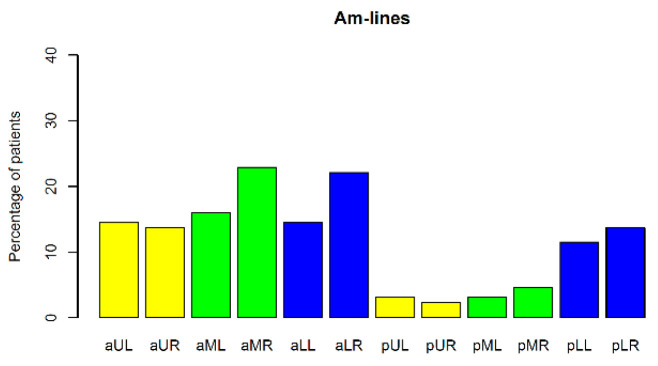
Incidence of Am-lines in individual lung fields. Lung fields: **aUL**—anterior upper left, **aUR**—anterior upper right, **aML**—anterior middle left, **aMR**—anterior middle right, **aLL**—anterior lower left, **aLR**—anterior lower right, **pUL**—posterior upper left, **pUR**—posterior upper right, **pML**—posterior middle left, **pMR**—posterior middle right, **pLL**—posterior lower left, **pLR**—posterior lower right.

Am-lines (Figure 4) occurred in 57 CF patients (44% of the study group). They were absent in the control group (Table 2). In most cases (30 individuals), a patient presented with one or two Am lines. The maximum number of Am-lines found was 20 in one case. The incidence of Am-lines in individual lung fields is shown in Figure 10.

#### 3.1.2. Pleural Line Abnormalities

**Figure 11:**
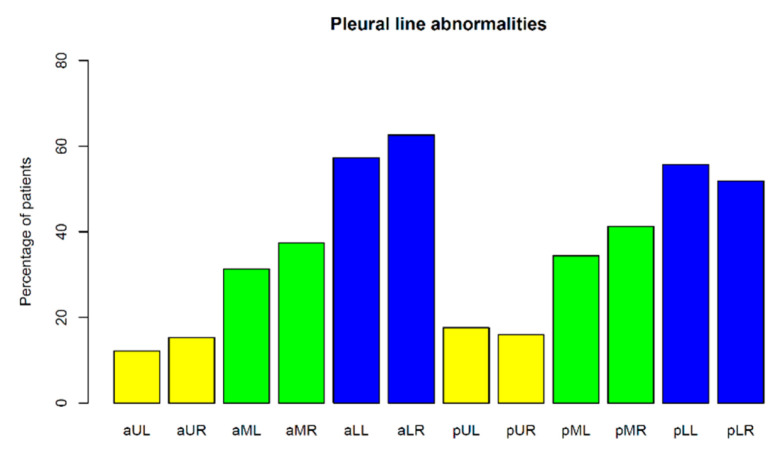
Incidence of pleural line abnormalities in individual lung fields. Lung fields: **aUL**—anterior upper left, **aUR**—anterior upper right, **aML**—anterior middle left, **aMR**—anterior middle right, **aLL**—anterior lower left, **aLR**—anterior lower right, **pUL**—posterior upper left, **pUR**—posterior upper right, **pML**—posterior middle left, **pMR**—posterior middle right, **pLL**—posterior lower left, **pLR**—posterior lower right.

Pleural line abnormalities occurred in ninety-five CF patients (73% of the study group) and in only five healthy children (16% of the control group) (Table 2). In the study group, the following pleural line abnormalities were found: irregularity in eighty-four children (64%), fragmentation in sixty-five (49%), blurring in sixty-two (47%), ragging in forty-three (33%) and thickening in four (3%) (Figure 5). These findings were reported more frequently in the lower than in the upper lung fields (Figure 11). The more severe the CF lung disease, the larger the surface of the lungs covered with affected pleura. In forty-six patients (35%), marked pleural line abnormalities were present in at least one lung field, among whom 14 patients had abnormalities in only one field; however, one patient had 10 affected lung fields. In the control group, a blurred pleural line was present in four patients (13%) and an irregular line presented in only one (3%). These abnormalities were minimal and occurred pointwise (over a limited area—1 to 2 cm^2^).

Statistical analysis revealed a significant association between the incidence of pleural line abnormalities and numerous B-lines (*p* < 0.0001). In 35 cases (97%), numerous B-lines were accompanied by pleural line abnormalities, while among children with no pleural line abnormalities, only one (3%) presented with numerous B-lines. Finally, patients with pleural line abnormalities, compared to patients without these findings, had significantly more lung fields with both single and numerous B-lines (7.10 ± 2.65 vs. 4.10 ± 2.68, *p* < 0.0001 and 0.72 ± 1.23 vs. 0.06 ± 0.33, *p* = 0.0002, respectively).

#### 3.1.3. Consolidations

Small consolidations (Figure 6) were found in eighty-four (64%) CF children and only in two (6%) healthy children (Table 2). In the control group, they were singular (one or two) and did not exceed 4 mm. In the study group, small consolidations were slightly bigger (up to 10 mm). Their number was greater in the anterior than in the posterior lung fields (*p* < 0.0001) and in the lower than in the middle and/or upper fields (*p* < 0.0001). They were equally frequent and numerous on both sides.

Major consolidations (Figure 7) were found in 38 (29%) CF patients but not in healthy children (Table 2). The size of consolidation was measured in two dimensions. These dimensions were multiplied to calculate the approximate cross-sectional area. The mean cross-sectional area of all consolidations was 9.03 cm^2^, with a standard deviation (SD) of 15.36 cm^2^. The considerable value of the SD results from the extensive range of area measurements. In some patients with advanced CF lung disease, the area exceeded 20 cm^2^ (Figures S2 and S3).

A total of 84 major consolidations was reported, including 67 with air bronchogram/air trapping. Vascularisation was possible to assess in 15 consolidations. In the case of the biggest consolidations, US signs of atelectasis and cirrhosis were present. Atelectatic lesions were characterised by the static air bronchogram (scantier than in acute inflammatory lesions) or the absence of air bronchogram, and by a tree-shaped vascular pattern with a distribution denser than the anatomical one. Cirrhotic lesions were characterised by a scanty static air bronchogram or the absence of air bronchogram, sparse or absent vascularisation, heterogeneous echogenicity and accompanying pleural line abnormalities.

#### 3.1.4. Pleural Fluid

Small amounts of pleural fluid (layer thickness: 2–7 mm) were reported in 32 patients (24% of the study group). The fluid appeared exclusively at the base of the lungs, most often in the costodiaphragmatic recesses. In the control group, physiological amounts of fluid (maximum 2 mm) were described in the costodiaphragmatic recesses in three children (9%) (Table 2). Fifteen CF patients (11%) had slightly larger amounts of fluid (3–7 mm) than children from the control group.

### 3.2. CF LUS Score Results

**Figure 12:**
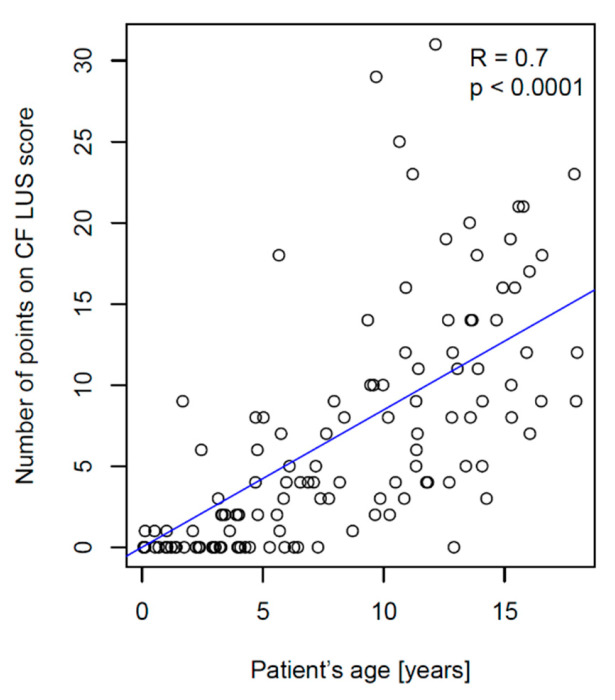
Correlation between the CF LUS score and the patient’s age. R—Pearson’s correlation coefficient; p—probability value.

The older the CF patient was, the greater the number of points they received on their CF LUS score—the correlation between age and CF LUS score was high (Figure 12). The distribution of the number of points on the CF LUS score in the study group is presented in Figure S4. Table S4 and Figure S5 show the distribution of the number of points in individual age groups.

#### 3.2.1. Ultrasound and Radiographic Score Comparison

**Figure 13:**
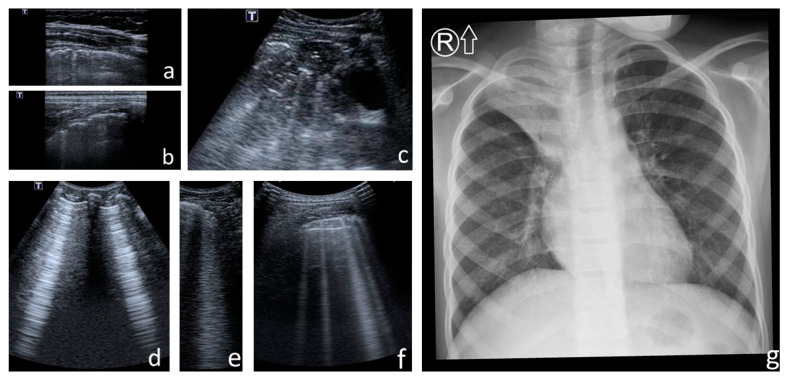
Exemplary collation of lung ultrasound (LUS) and chest X-ray (CXR) images. (**a**–**f**) **LUS—elements assessed with the CF LUS score:** (**a**,**b**) pleural line abnormalities; (**c**) consolidation covering the entire upper lung field; front and rear, with heterogeneous echogenicity and irregular static air bronchogram/air trapping (hyperechoic points, spots, and linear structures within the consolidation)—cirrhosis; (**d**) Am-lines; (**e**) confluent B-line; (**f**) numerous B-lines. (**g**) **CXR—elements assessed with the modified Chrispin–Norman score:** barrel chest, excessive transparency of the lung fields (overinflation of the middle lobe and left upper lobe), cirrhosis of the upper right lobe with atelectasis and air bronchogram, bilateral lesions consistent with bronchiectasis with wall thickening (linear peribronchial opacities including tram-track sign, and ring shadows). Total number of points on the CF LUS score—29, on the modified Chrispin–Norman score—17.

Major consolidations and lesions consistent with bronchiectasis were visualised both with LUS and CXR (Figure 13). On the one hand, CXR did not reveal small consolidations, pleural line abnormalities or small amounts of pleural fluid. On the other hand, LUS did not reveal the lesions separated from the probe with properly aerated lung or bone, i.e., located mainly in the perihilar and subscapular areas.

**Figure 14:**
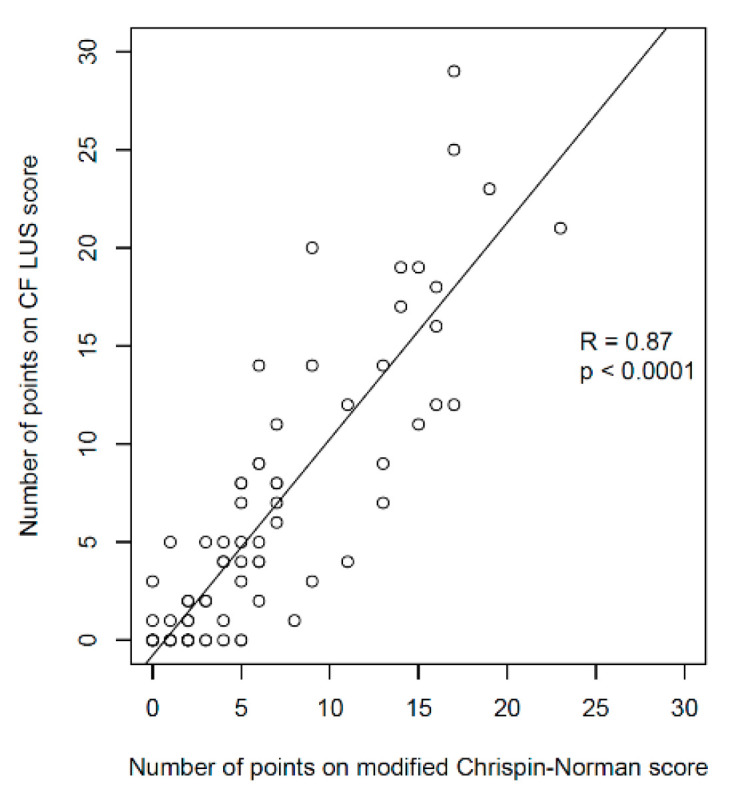
Correlation between the ultrasound and radiographic scores.

A very strong correlation was found between the number of points on the CF LUS score and the number of points on the modified Chrispin–Norman score (Figure 14). The distribution of the number of points was very similar for the two scores—both in terms of the number of patients in individual point compartments (Figure S6) and the number of points in individual age groups (Figure S7). In single patients with advanced CF lung disease, there was a higher number of points on the CF LUS score than on the modified Chrispin–Norman score. Figure 13 shows an exemplary collation of LUS and CXR images and the elements assessed with both scores.

#### 3.2.2. Correlation between CF LUS Score and PFTs Results

Based on spirometric results, two out of seventy-seven patients (3%) suffered from a severe form of CF lung disease (FEV1 predicted < 40%), five (6%) suffered from a moderate form (FEV1 pred. 40—70%) and seventy (91%) suffered from a mild form of CF lung disease (FEV1 pred. > 70%).

**Figure 15:**
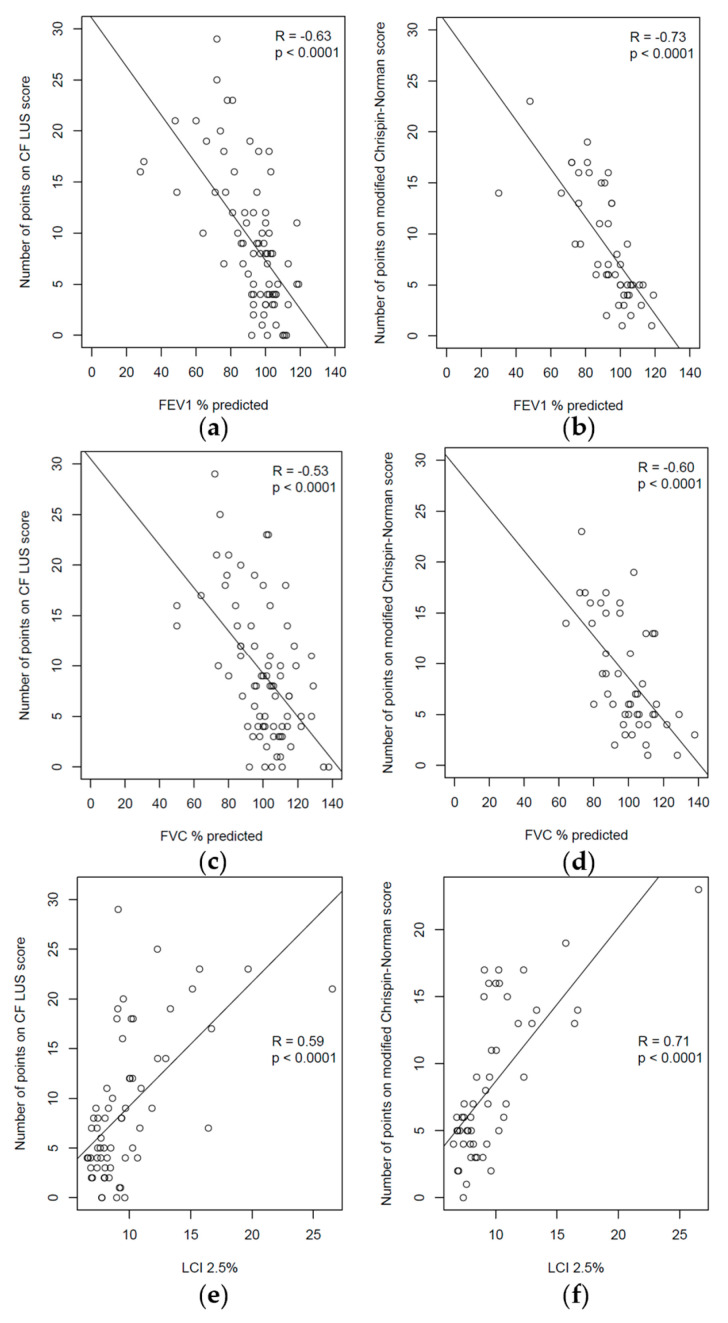
Correlations between the ultrasound and radiographic scores and the PFTs results: (**a**) CF LUS score and FEV1; (**b**) modified Chrispin–Norman score and FEV1; (**c**) CF LUS score and FVC; (**d**) modified Chrispin–Norman score and FVC; (**e**) CF LUS score and lung clearance index (LCI); (**f**) modified Chrispin–Norman score and LCI.

A strong, negative correlation was found between the number of points on the CF LUS score and the spirometry test results (Figure 15a,c). A strong, positive correlation was determined between the number of points on the CF LUS score and the MBNW test results (Figure 15e). It is worth noting that the correlation between the numbers of points on the modified Chrispin–Norman score and the PFTs results was slightly higher (Figure 15b,d,f).

#### 3.2.3. Correlation between CF LUS Score and Microbiological Status

In sixty-three CF patients (48% of the study group), respiratory tract infections with CF pathogens were diagnosed, out of which: seventeen (27%) were chronic, eleven (18%) were intermittent, thirty-three (52%) had been previously reported, and two (3%) were first-time infections. The following pathogens were cultured: in the majority of cases (fifty-three; 40% of the study group)—*Pseudomonas aeruginosa*, in seven (5%)—*Stenothrophomonas maltophilia*, in five (4%)—methicillin-resistant *Staphylococcus aureus* (MRSA), and in single patients—other *Pseudomonas* strains (*stutzeri, putida, fluorescens, alcaligenes,* spp.), *Achromobacter xylosoxidans*, *Burkholderia multivorans*, *Burkholderia cepacia*, *Pandorea pulmonicola* and *Comamonas testosteroni*. Three children were diagnosed with infections caused by several pathogens simultaneously.

**Figure 16:**
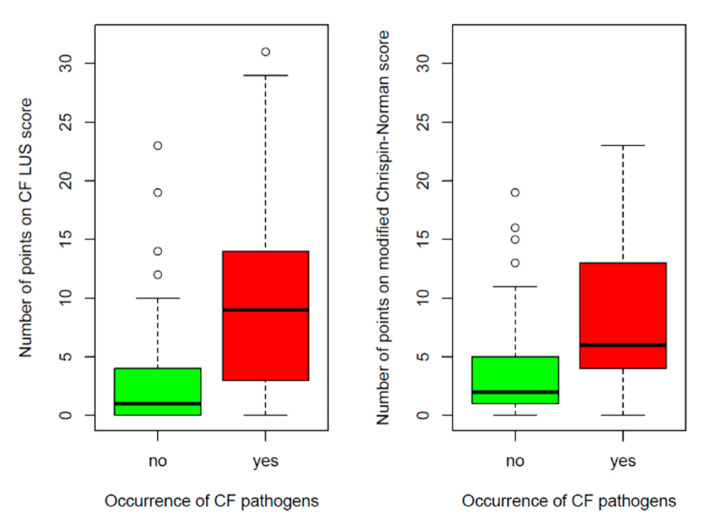
Number of points on the ultrasound and radiographic scores depending on the occurrence of CF pathogens.

**Figure 17:**
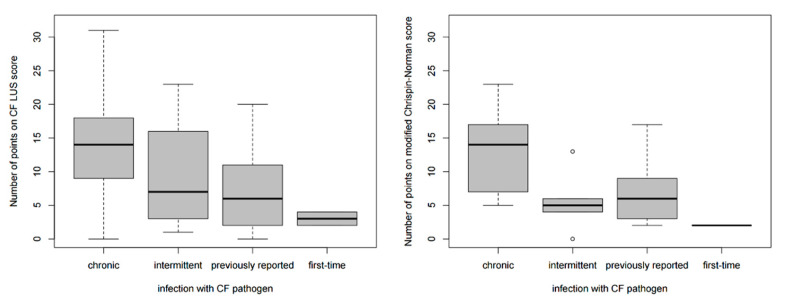
Number of points on the ultrasound and radiographic scores depending on the type of infection with CF pathogens.

Patients with CF pathogens had a higher number of points on the CF LUS score than patients without these bacteria (9.64 ± 7.59 vs. 2.97 ± 4.58, *p* < 0.0001) (Figure 16). The number of points on the CF LUS score differed significantly depending on the type of infection (*p* = 0.0103, Kruskal-Wallis test). The highest scores were found in subjects with chronic infection and the lowest in subjects with first-time infections. There were no significant differences between intermittent and previously reported infections (Figure 17).

**Figure 18:**
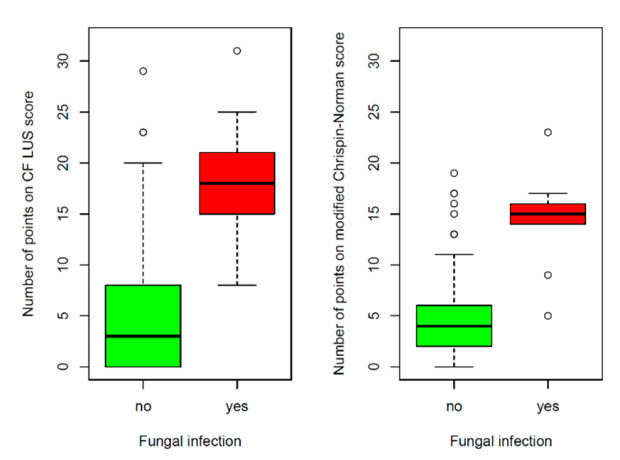
Number of points on the ultrasound and radiographic scores depending on the occurrence of fungal infection.

In twelve CF patients (9% of the study group), fungal infections of the respiratory tract were diagnosed (two in actual culture and ten previously reported). Children with fungal infections had a higher number of points on the CF LUS score than children without such an infection (18.25 ± 6.08 vs. 4.96 ± 5.89, *p* < 0.0001) (Figure 18).

It is worth noting that the comparison of the radiographic score with microbiological tests results was similar. Higher numbers of points on the modified Chrispin–Norman score were noted both in patients with CF pathogens (8.32 ± 5.64 vs. 3.96, *p* < 0.0001) and in patients with fungal infections (14.33 ± 6.08 vs. 4.88 ± 4.57, *p* < 0.0001) (Figure 16 and Figure 18). The number of points on the modified Chrispin–Norman score differed significantly depending on the type of infection (*p* = 0.0211, Kruskal–Wallis test) (Figure 17).

#### 3.2.4. Reference of Selected US Signs to the Results of Other Diagnostic Tests

##### Numerous B-Line Artefacts

Patients with numerous B-lines, compared to patients without these artefacts, were older (mean age in years: 11.99 ± 3.81 vs. 5.99 ± 4.86, *p* < 0.0001) and received more points on the CF LUS score (13.61 ± 7.24 vs. 3.37 ± 4.48, *p* < 0.0001) and on the modified Chrispin–Norman score (10.45 ± 4.56 vs. 4.46 ± 4.81, *p* < 0.0001). In these patients, infections with CF pathogens and with fungi were found more often (72% vs. 39% and 25 vs. 3%, respectively, *p* < 0.0001).

##### Am-Line Artefacts

**Figure 19:**
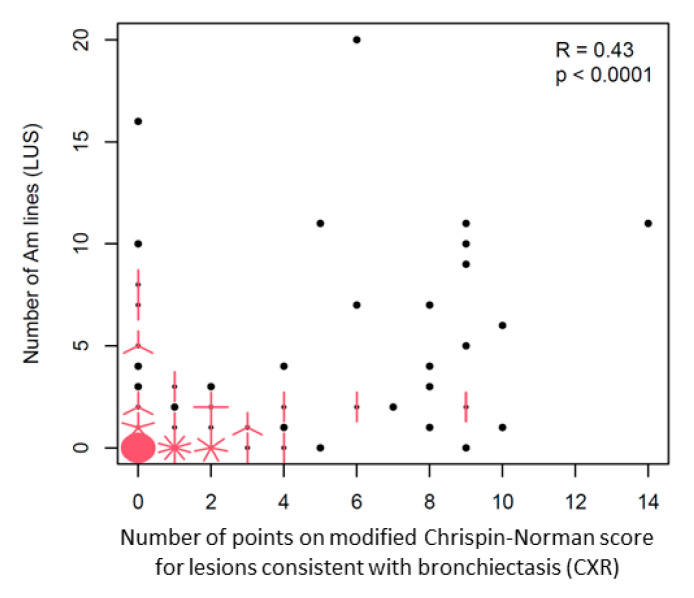
Correlation between lesions consistent with bronchiectasis in LUS and CXR. The black dot represents a patient with a given number of Am lines and number of points on the modified Chrispin-Norman score. Each red line represents another patient with the same numbers of both variables. The red circle is a large number of collected red lines (meaning: a lot of patients received 0 points for both variables).

Statistical analysis showed a moderate correlation between the number of Am-lines and the number of points on the modified Chrispin–Norman score obtained for the lesions consistent with bronchiectasis on CXR (i.e., bronchial line shadows and ring shadows) (Figure 19). Furthermore, patients with Am-lines, compared to patients without these artefacts, were older (mean age in years: 11.30 ± 4.24 vs. 4.80 ± 4.18, *p* < 0.0001) and received a higher number of points not only on the ultrasound score (14.30 ± 6.27 vs. 3.20 ± 4.12, *p* < 0.0001), but also on the radiographic score (9.31 ± 5.61 vs. 2.89 ± 3.04, *p* < 0.0001). They were also more often diagnosed with CF pathogens and fungal infections (72% vs. 30% and 21% vs. 0%, respectively, *p* < 0.0001). Meanwhile, children who received points on the modified Chrispin–Norman score for the lesions consistent with bronchiectasis, compared to children who received no points, were older (9.0 ± 4.9 vs. 6.5 ± 5.4, *p* = 0.0035) and received a higher number of points on the ultrasound score (7.80 ± 7.44 vs. 4.80 ± 6.43, *p* = 0.0036) and on the radiographic score (7.80 ± 5.41 vs. 1.30 ± 1.43, *p* < 0.0001). They were more often diagnosed with fungal infections (16% vs. 3%, *p* = 0.0175), but there was no difference in terms of the incidence of CF pathogens.

##### Small Consolidations

Small consolidations were revealed with LUS but were not visible on CXR. Patients with small consolidations did not differ significantly from those without consolidations in terms of age, the number of points on the ultrasound and radiographic scores or the occurrence of CF pathogens.

##### Major Consolidations

**Figure 20:**
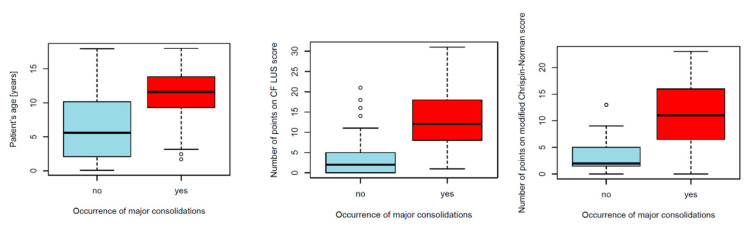
Comparison of patients with and without major consolidations.

In the locations where LUS revealed major consolidations, the following lesions were described on CXR: small parenchymal consolidations, mottled shadows consistent with mucous plugs, and extensive parenchymal consolidations/large soft shadows with or without atelectasis and cirrhosis. Children who had major consolidations, compared to children without major consolidations, were older (mean age in years: 10.9 ± 4.4 vs. 6.3 ± 5.1, *p* < 0.0001) and received more points on the ultrasound score (13.3 ± 7.3 vs. 3.3 ± 4.4, *p* < 0.0001) and on the radiographic score (11.1 ± 5.7 vs. 3.4 ± 3.1, *p* < 0.0001) (Figure 20). They were more often diagnosed with CF pathogens and fungal infections (71% vs. 39%, *p* < 0.0015 and 25% vs. 2%, *p* < 0.0001, respectively).

##### Pleural Fluid

**Figure 21:**
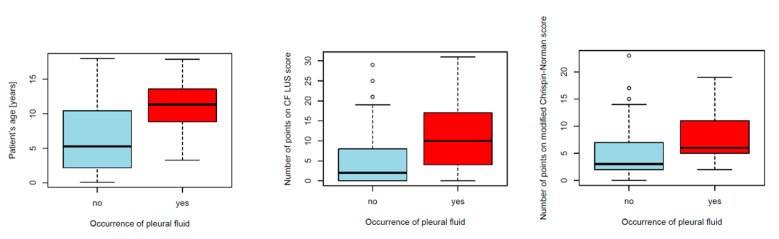
Comparison of patients with and without pleural fluid.

Small amounts of pleural fluid were visualised in 24% of the study group with the use of LUS but not with the use of CXR. Compared to children without pleural fluid, children with pleural fluid were older, received more points on the ultrasound and radiographic scores (Figure 21) and manifested an increased incidence of numerous B-lines and CF pathogens.

### 3.3. Interobserver Study

#### 3.3.1. Interobserver Agreement for the Assessment of Individual US Signs

The values of the ĸ coefficient for assessing the incidence of particular US signs in individual lung fields and pleural cavities ranged from 0.26 to 1.00, except for Z-lines, for which ĸ was significantly lower (0.05–0.53) (Tables S5–S12). The mean strength of agreement was ‘very good’ for the assessment of most US signs, ‘good’ for one, ‘moderate’ for two, and ‘fair’ for one (Table 3). The best agreement (both in terms of the mean value and for individual lung fields) was obtained for the evaluation of numerous B-lines (ĸ = 0.95) and major consolidations (ĸ = 0.94). Furthermore, in patients with major consolidations, both sonographers obtained very similar results for the total cross-sectional area of consolidations (Figure S8).

#### 3.3.2. Interobserver Agreement for the Evaluation of CF Lung Disease Severity

**Figure 22:**
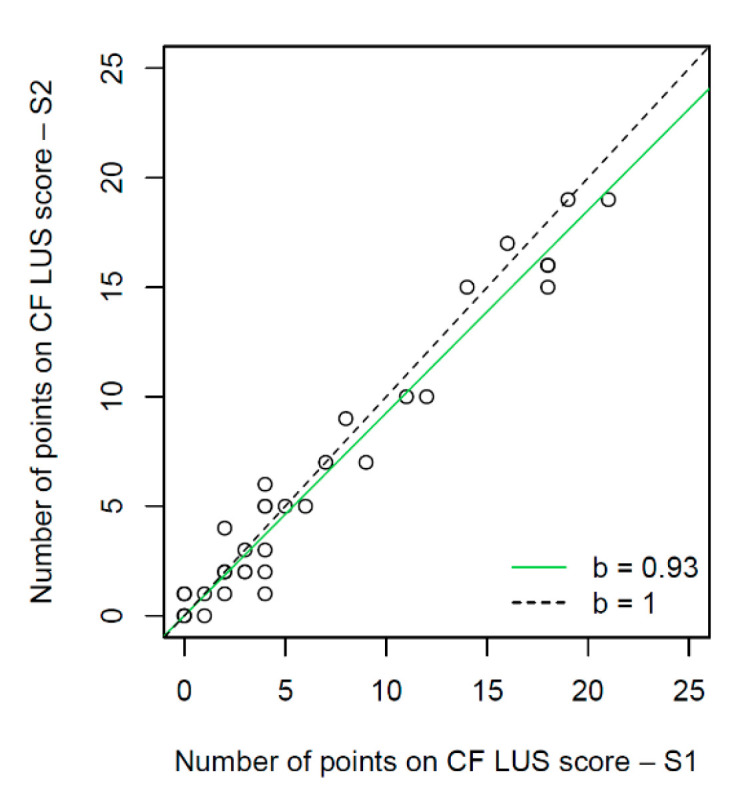
Comparison between the number of points on the CF LUS score obtained by two sonographers. S1—first sonographer, S2—second sonographer, b—slope of simple linear regression.

The number of points on the CF LUS score was identical for both investigators for seven patients, while the remaining children were assessed very similarly (Figure 22). The interval estimate of the slope of the regression line [0.89; 0.97] at the 95% confidence level indicated that the results on the ultrasound score of the second sonographer were slightly lower compared to the first sonographer. The concordance correlation coefficient for the number of points on the CF LUS score obtained by the two researchers was 0.98 (95% CI [0.96; 0.99]). The correlation coefficients for age with the number of points on the CF LUS score did not vary significantly between sonographers (R = 0.67 vs. R = 0.66, *p* < 0.0001).

## 4. Discussion

### 4.1. Specificity of the Study Group

The study group was a valuable group of patients who has remained under the holistic care of a multidisciplinary team, in most cases, from the first months of life, in accordance with the European standards [4]. During regular follow-up visits, diagnostic tests are performed, medical, nursing, dietary, physiotherapeutic and psychological consultations are held and social support is provided. In such conditions, dietary and respiratory physiotherapy errors are identified quickly and corrected before being consolidated by the patient/patient’s caregiver. The appearance of CF pathogens in the respiratory tract or worsening of lung function on the PFTs is also noticed at a very early stage—most often before the development of clinical symptoms. Such proceedings allow for the number of pulmonary exacerbations to be significantly reduced and lung disease progression to be decelerated. They also cause most children under the CF Centre’s care to retain in optimal condition in terms of their disease.

### 4.2. The Meaning of US Signs Found in Children with CF

#### 4.2.1. I-Line and Z-Line Artefacts

This study showed that in CF patients, I-lines and Z-lines were bigger in number, occurred more frequently and on a larger lung surface than in healthy children. To the authors’ best knowledge, there is no research documenting an increased number of these artefacts in other diseases. They are considered to be a component of a normal ultrasound image of the lungs, although their significance and formation mechanism are unclear.

#### 4.2.2. B-Line Artefacts

In the presented study, B-lines were divided in terms of their number into single (≤3) and numerous (≥4) per scan with the transverse (in relation to the body axis) application of a transducer. The same division was used in a paper assessing pulmonary fibrosis in adult patients with interstitial diseases [34]. This approach differs from the more commonly used one, in which with the longitudinal application of a probe, up to two B-lines per scan are considered a normal lung image, and three or more B-lines indicate abnormality. This classic division was created by Lichtenstein for intensive care unit patients who underwent LUS with the longitudinal application of a transducer in selected locations [49,50]. In the presented work, the entire surface of the lungs was examined in clinically stable patients. The transverse application was treated as the basic one, and the longitudinal application was used in case of doubt in order to view the assessed area in a different projection. In the authors’ opinion, this examination method makes it possible to evaluate the entire available area of the lungs more efficiently because one scan in the transverse probe’s application covers a larger lung area than in the longitudinal application, which includes the ribs and their acoustic shadows. The applied division has proven to be valid for the control groups both in the presented work and in the cited study on pulmonary fibrosis—none of the control subjects had more than three B-lines per scan.

In this study, single B-lines were found in 23 healthy children (72% of the control group), mainly in the lower lung fields and less often in the middle ones. Meanwhile, in the study group, they were found in 130 children (99%) in different lung fields. Single B-lines are considered to be part of a normal LUS image. However, in CF patients, their incidence on a much larger lung area than in healthy children may be caused by progressive interstitial changes (chronic inflammation and fibrosis).

Numerous B-lines are considered to be a component of an abnormal LUS image. They occur in many lung diseases in which the pathological process takes place in the interstitium, alveoli and pleura [51,52]. Similarly to other diseases, in cystic fibrosis, a correlation between the advancement of the disease process and the number and distribution of B-lines was observed. The more severe the CF lung disease, the more numerous B-lines occurred over a larger area of the lungs. Authors of other studies on LUS applied to CF patients made analogous observations and also included this sign in their ultrasound scores (Table S13) [22,23,24,26]. Information about the intensity and distribution of B-lines has some diagnostic value. However, observation of these changes over time is of the utmost importance for the clinicians treating CF patients. This remark is based on the authors’ clinical experience and confirmed by the reports of other researchers [22,23].

Confluent (or coalescent) B-lines indicate the local intensity of lesions. In the presented study, they were found in only seven patients (5% of the study group). Strzelczuk-Judka et al. reported the incidence of confluent B-lines in 20 out of 48 patients enrolled in the study (42%). It seems that the reason for this discrepancy is different age range of patients (5 weeks—18 years vs. 5–18 years) or a more restrictive exclusion criteria in the presented study (PEx, cough exaggeration and/or other clinical symptoms of acute respiratory tract infection during the 6 weeks preceding the examination).

Ciuca et al. proposed a partially different interpretation of the numerous and confluent B-lines [26]. After comparing LUS with CT images, they concluded that numerous B-lines might represent not only interstitial inflammatory lesions but also small bronchiectasis in CF patients. The authors suggested that confluent B-lines might correspond not only to the inflammatory process in the interstitium and alveoli but also to mucous plugs, bronchial wall thickening and bronchiectasis. This lack of specificity of ultrasound artefacts prompts researchers to be cautious in their interpretations. However, it does not change the fact that the semi-quantitative assessment of selected US signs in appropriately constructed scores enables the evaluation of CF lung disease, which correlates with the assessment of the CXR and CT scores (Table S13).

#### 4.2.3. Am-Line Artefacts

Am-lines have been described so far in only one study involving patients with interstitial lung diseases [34], in whom the presence of these artefacts correlated with the presence of subpleural cysts and emphysematous bullas (honeycombing) on chest CT scans. Am-lines combine the features of A-lines and B-lines. The mechanism of formation has yet to be elucidated and requires further research. Most likely, it is a reverberation artefact—the result of multiple reflections of ultrasound waves between two border surfaces. The first border surface would be the pleural line, and the second the wall of emphysematous bulla/cyst or bronchiectasis. Buda et al. observed Am-lines in 57 patients (44% of the study group). These artefacts were an essential element of the authorial LUS score designed to evaluate pulmonary fibrosis severity. The optimal comparison method for Am-line artefacts should be CT (the gold standard for assessing bronchiectasis). However, at the time of conducting the research for this study, chest CT was not yet included in the routine diagnostics of clinically stable patients at the CF Centre of the Institute of Mother and Child in Warsaw. For this reason, the incidence of Am-lines has been compared to the incidence of CXR lesions consistent with bronchiectasis. They were defined as bronchial line shadows and ring shadows on the modified Chrispin–Norman score [35,36]. Statistical analysis showed a moderate correlation between the number of Am-lines and the sum of points on the modified Chrispin–Norman score for the above-described lesions. The unsatisfactory correlation is a consequence of the different limitations of the compared imaging methods. On the one hand, CXR reveals lesions in locations inaccessible for ultrasounds (perihilar and subscapular areas). On the other hand, LUS reveals lesions that cannot be visualised with CXR. Patients with lesions consistent with bronchiectasis in LUS and CXR differed significantly from patients without such lesions. They were older, received more points on the ultrasound and radiographic scores and had more frequent fungal infections. Additionally, in children with Am-lines, CF pathogens were more common. Therefore, it should be concluded that the incidence of Am-lines is one of the important elements in the evaluation of the advancement of CF lung disease using LUS.

Among the publications describing the use of LUS on patients with CF, only one mentioned LUS lesions that, according to the authors, corresponded to bronchiectasis on CT scans [26]. The following US signs were listed and interpreted: 1. more than three B-lines and one coalescent B-line—interstitial inflammation or small bronchiectasis; 2. more than two coalescent B-lines—alveolo-interstitial inflammation or mucus plugging with loss of aeration; 3. bronchial wall thickening or subpleural consolidations <10 mm—small atelectasis or cystic bronchiectasis with mucous plugging. The interpretation of artefacts can be dependent on the ultrasound apparatus and the preset used for examination [17]. In order to find the reason for the discrepancies in the description of lesions consistent with bronchiectasis, a multicentre study should be conducted.

#### 4.2.4. Small Consolidations

The clinical significance of small consolidations remains unclear. Single small consolidations are found in healthy children. It is known from a few available publications that in healthy children, small consolidations usually do not exceed 5 mm in the largest dimension. Since consolidations ≤10 mm cannot be visualised with CXR, most authors currently use this criterion for small consolidations [23,53,54,55,56,57,58]. This US sign is described in patients with various respiratory diseases—pneumonia [54,55,56,57], bronchiolitis [58,59,60,61], pulmonary embolism [62,63] and acute respiratory distress syndrome [64]. Shah et al. demonstrated an increase in the specificity of diagnosing pneumonia (assessed with LUS vs. CXR) from 86 to 97% when considering only consolidations > 10 mm [65]. In contrast, in a few children with pneumonia, who presented with clinical symptoms and elevated inflammatory markers, CXR did not show any abnormalities; only small consolidations were visualised with LUS [65,66]. In a recently published study analysing the importance of this US sign in children with symptoms of respiratory tract infection and suspected pneumonia, small consolidations were found in 62 out of 188 patients [53]. In a subgroup of 39 children with isolated small consolidations, 21% had pneumonia confirmed by the means of CXR, and 41% were prescribed antibiotic therapy due to suspected pneumonia. Nonetheless, two other papers have highlighted that revealing small consolidations may lead to the overuse of antibiotics in children with lower respiratory tract infections [54,67]. According to some authors, small consolidations are related to the viral aetiology of the infection [68,69].

The above reports indicate that small consolidations may be of clinical significance in some patients and should be interpreted in relation to the entire clinical picture. It is also worth performing a control LUS examination to assess the evolution of these lesions. In the presented study, single (one to two), minor (max. 4 mm) consolidations were found in only two healthy children. In contrast, consolidations ≤10 mm were visualised in 84 CF patients (64% of the study group). Other authors found this US sign in a similar percentage of children with CF (58%) [23].

The presence of small consolidations in CF patients is probably related to the occlusion of the lumen of terminal parts of the airways with thick mucus and small areas of parenchymal aeration dysfunction. In the presented study, children with CF who had isolated small consolidations did not differ significantly in terms of any verified parameter. This was a cross-sectional study and therefore we did not control the evolution of these lesions. However, in the authors’ personal experience, some small consolidations may appear in different locations in control LUS. In some cases, in place of the previously visualised small consolidations, major consolidations are found during PEx. These observations are consistent with the results presented in existing publications on the use of CT in lung imaging of children with CF [26,70]. It has been demonstrated that peripheral mucus plugs cause the formation of small foci of inflammation, which may lead to exacerbations of the disease, and that localising such lesions in CT may precede clinical symptoms or the deterioration of lung function shown in PFTs [7,71,72]. This is one of the arguments in favour of performing regular imaging tests as an element of annual follow-up examinations.

Small consolidations were included in the CF LUS score and the ultrasound scores developed by other authors for patients with CF (Table S13).

#### 4.2.5. Major Consolidations

Major consolidations revealed using LUS correspond to lesions in the lung parenchyma, which are most often visible both on CXR and CT. Therefore, their interpretation is not controversial. In the presented study, major consolidations were found in 38 CF children (29%). Similar results were reported in other studies involving paediatric patients (17–32%) [23,26]. In contrast, in heterogenous study groups which included children and adults, the proportions of patients with major consolidations were higher (63–67%) [24,25]. This research showed that children with major consolidations differed significantly from children without this US sign in terms of all assessed parameters. They received more points on radiographic and ultrasound scores and were more often diagnosed with CF pathogens and fungal infections of the respiratory tract. In a study comparing the diagnostic value of LUS and CXR in relation to chest CT as a reference method in PEx, LUS was significantly more sensitive and specific than CXR in detecting consolidations (90 vs. 73% and 95 vs. 60%, respectively) [25]. Major consolidations are a crucial element of the CF LUS score, and they were also included in all other ultrasound scores developed for CF patients (Table S13).

#### 4.2.6. Pleural Line Abnormalities

We found pleural line abnormalities in several children from the control group (blurred in four patients and irregular in one). These US signs were minimal, occurred pointwise and occupied a limited area. The abnormalities could be residual lesions after respiratory tract infections (one boy had a history of pneumonia a year before, and in the remaining cases, respiratory tract infections were diagnosed a few months before enrolment in the study).

In the presented study, pleural line abnormalities occurred in most patients with CF (95 cases—73%). There was a significant association between the incidence of pleural line abnormalities and the presence of numerous B-lines. These results suggest that the chronic inflammatory process leading to fibrosis of the lung interstitium and the chronic inflammatory process leading to fibrosis of the pleura are related to each other. The description of pleural line abnormalities was not found in most publications on the use of LUS in CF [22,24,25,26]. In one study, this US sign was omitted because the sensitivity of CT in detecting pleural lesions is lower than that of LUS [26]. In another study, only the convex transducer was used, with which solely severe pleural lesions can be visualised [25]. Only one study included pleural line abnormalities in the ultrasound score, but they were found in only one out of forty-eight CF patients [23].

#### 4.2.7. Pleural Fluid

Pleural fluid was not included in the CF LUS score since patients enrolled in the study had only small amounts of clear fluid at the bases of their lungs. The inclusion of this parameter in the ultrasound score in future research is definitely worth considering, especially in patients with PEx. In the study group, pleural fluid occurred more frequently than in the control group (24% vs. 9%) and in slightly larger amounts (layer thickness: max. 7 mm vs. max. 2 mm). CF patients with pleural fluid compared to patients without this sign differed significantly in terms of age, the number of points received on both scores (CF LUS and modified Chirspin-Norman) and the presence of CF pathogens. The underlying causes of this phenomenon are probably chronic inflammation of the respiratory system and pleura, and recurrent infections, which often lead to PExs.

In one other study, the presence of pleural fluid was included in the ultrasound score [23]. The thickness of the layer in mm was not given, but it was determined that it occurred in the costodiaphragmatic recesses or along the chest wall.

### 4.3. Ultrasound Scores

The characteristics of various ultrasound scores used to evaluate the lung disease severity are presented in Table S13. Compared to the score developed by Strzelczuk-Judka et al. [23], CF LUS score is characterised by the following:

- Significantly higher correlation with the radiographic score (R = 0.87 vs. 0.52);
- Significantly higher correlation with patient’s age (R = 0.7 vs. 0.12);
- Greater diversification of the number of points depending on the patient’s age;
- Higher maximum number of points obtained in the study group (31 vs. 16).

The above-described advantages of this score may result from the consideration of single B-lines (if they were present in all pulmonary fields in a given area); greater point nuance of signs, such as small consolidations and pleural line abnormalities; and consideration of Am-lines (a sign consistent with bronchiectasis) (Table 1).

Compared to the score developed by Ciuca et al. [26], the presented score has a significantly lower correlation with LCI (R = 0.59 vs. 0.8) and similarly correlates with FEV1% pred. (R = −0.63 vs. −0.65). The obtained results cannot be directly compared with those published by Peixoto et al. due to relying on different statistical methods [24].

### 4.4. Interobserver Study

The weakest interobserver agreement was found for the assessment of Z-lines (mean value of ĸ = 0.34), which are of unknown clinical significance. Other studies evaluating the agreement between sonographers have not considered these artefacts. For the assessment of the remaining US signs, the interobserver agreement was higher than reported by other authors:

- B-lines: single—good (ĸ = 0.61), numerous—very good (ĸ = 0.95); in other authors’ works, the agreement for B-lines ranged between moderate and good (ĸ: 0.41—0.79) [73,74,75];
- Consolidations—very good (small—0.84, major—0.94); in other articles, agreement ranged between fair and moderate (0.34—0.59) [73,74,76,77];
- Pleural fluid—very good (0.81); in other publications, agreement was moderate (0.44–0.49) [73,74];
- Pleural line abnormalities—moderate (0.57); in other articles, agreement was fair (0.23) [73,74].

The agreement for the assessment of lung disease severity on CF LUS score was very good (concordance correlation coefficient = 0.98). A slightly lower agreement (ĸ: 0.83–0.86) was obtained by researchers assessing the advancement of bronchiolitis lesions on various ultrasound scores [58,78]. In contrast, interobserver agreement for assessing the advancement of COVID-19 lesions in adults [76] and assessing the degree of lung aeration in children in the intensive care unit [79] was fair to moderate.

There may be several reasons for the higher agreement between the sonographers in the presented work compared to most of the available publications. Firstly, before starting the interobserver study, the definitions of US signs were discussed, and the method of performing LUS was standardised, including the patient’s body position during the examination and the settings of the apparatus. Secondly, these examinations were conducted in clinically stable patients; thus, these situations were devoid of the time pressure typical for working with patients in emergent conditions. Thirdly, each of the clinicians in the presented research performed LUS personally, while in some of the cited studies, the images were recorded and later analysed by another sonographer. Fourthly, LUS in this study was performed with two transducers on each patient (linear and convex), and there are reports confirming that the type of transducer used may affect the interpretation of results and the interobserver agreement [80].

### 4.5. Strengths and Limitations of the Study

The main limitation of this work is the lack of comparison of LUS results with the results of chest CT, which is considered to be the gold standard for the assessment of most pathological changes observed in the course of CF lung disease. This lack of reference to CT results from the fact that at the time of performing this study, CT was not part of a regular evaluation of clinically stable paediatric patients at the Cystic Fibrosis Centre of the Institute of Mother and Child in Warsaw. The comparison of lesions found in LUS and in CT, as well as the correlation between the scores based on both imaging methods, are presented in two publications [24,26] (Table 4). In one study, selected LUS and CXR signs were compared in relation to CT, but the scores were not used [25].

Another limitation of this work is the fact that it is a cross-sectional study. It did not aim to observe the evolution of lesions over time, which is important in the case of a chronic disease. The intention of the authors was, first of all, to elaborate a description of the ultrasound image of the lungs in children in the stable phase of CF lung disease, so that it would then be a reference point in the case of PExs and complications.

To avoid visualisation of residual lesions from previous infections, clear exclusion criteria were used—6 weeks prior to the examination, neither symptoms of PEx nor acute respiratory tract infections could be present. Such restrictive criteria were not found in any of the other works. Other advantages of the presented study include:

- Comparison of LUS results in CF patients with those of healthy children (although the control group could have been larger);
- Large study group (over two times larger than the biggest group among the compared studies), also including the youngest children—patients diagnosed with CF in the second month of life;
- Comparison of LUS results not only with CXR results (along with the correlation of the scores) but also with the results of PFTs (such comparisons are also presented in two other studies [24,26]) and with the microbiological status of the respiratory tract;
- Inclusion of Am-lines in assessment, artefacts which may be consistent with bronchiectasis [34];
- Conducting an interobserver study on a large proportion of the study group (29%), which enabled us to check the reproducibility of LUS in terms of individual US signs and in terms of the evaluation of the lung disease severity using the ultrasound score.

### 4.6. Summary

To sum up, LUS is a relatively cheap, commonly accessible, ionising radiation-free and reproducible imaging method worth applying to children with CF. Currently, there is insufficient evidence to propose the replacement of CXR with LUS for annual follow-ups in clinically stable patients [81]. However, since the assessment of lung disease severity based on ultrasound scores corresponds to the assessment based on CXR and CT scores, PFTs’ results and microbiological tests results, it is worth including LUS in the annual workup, starting with CF diagnosis in infants. It will provide a valuable reference point for the evaluation of the respiratory system in case of PExs, complications, and for treatment monitoring. It is expected that LUS will reduce the number of CXRs performed between routine check-ups. Such a proceeding has already been partially implemented at the CF Centre of the Institute of Mother and Child in Warsaw. It is a great aid in everyday clinical practice, and also in differential diagnosis of dyspnoea and chest pain.

In the authors’ opinion, future studies should concentrate on examining the diagnostic value of LUS in diagnosing and treating PExs and complications in CF patients. Another research area that could lead to further standardisation and objectification of the obtained results is the attempted use of artificial intelligence in the assessment of ultrasound images [17,82,83,84].

## 5. Conclusions

1. Lung ultrasound is a valuable test in the evaluation of CF lung disease severity. It is comparable with methods routinely used during check-ups of patients with clinically stable CF, i.e., chest radiograph, pulmonary function tests and microbiological tests of the respiratory system.
2. Lung ultrasound and chest radiograph should be treated as complementary diagnostic procedures as each of these imaging methods has different advantages and limitations.
3. Lung ultrasound is a method of high interobserver reproducibility in the evaluation of lung disease severity in children with CF, when appropriately standardised.

## Figures and Tables

**Table 1:** Cystic fibrosis lung ultrasound Score (CF LUS Score).

Finding	Degree of Intensity		
**small consolidations (≤10 mm)**	**≤1**	**2–3**	**≥4**
Right Lung—Anterior Area (RL-A)	0	1	2
Right Lung—Posterior Area (RL-P)	0	1	2
Left Lung—Anterior Area (LL-A)	0	1	2
Left Lung—Posterior Area (LL-P)	0	1	2
**major consolidations (>10 mm)**	**absent**	**single ≤ 40 mm**	**≥2 or 1 > 40 mm**
RL-A	0	1	2
RL-P	0	1	2
LL-A	0	1	2
LL-P	0	1	2
**pleural line abnormalities**	**absent** **or minimal**	**present,** **not marked**	**marked** **or widespread**
RL-A	0	1	2
RL-P	0	1	2
LL-A	0	1	2
LL-P	0	1	2
**Am-line artefacts**	**absent**	**≤2**	**≥3**
RL-A	0	1	2
RL-P	0	1	2
LL-A	0	1	2
LL-P	0	1	2
B-line artefacts *	**≤3**	**confluent or ≥4** **in 1 of 3 lung fields**	**≥4 in most of the area (in at least 2 of 3 lung fields)**
RL-A	0 *	1	2
RL-P	0 *	1	2
LL-A	0 *	1	2
LL-P	0 *	1	2
* Additional 1 point is to be given if 1-3 B-line artefacts are present in all 3 lung fields in the area (max. 4 additional points).			

Maximum score—44 points.

**Table 2:** Ultrasound (US) signs in CF patients compared with healthy children.

US signs	Study Group (*n* = 131)		Control Group (*n* = 32)	
	Number of Children	Percentage	Number of Children	Percentage
I-lines	123	94%	17	53%
Z-lines	108	82%	15	47%
single B-lines	130	99%	23	72%
numerous B-lines	36	27%	0	0%
confluent B-lines	7	5%	0	0%
Am-lines	57	44%	0	0%
pleural line abnormalities	95	73%	5	16%
small consolidations	84	64%	2	6%
major consolidations	38	29%	0	0%
pleural fluid	32	24%	3	9%

**Table 3:** Interobserver agreement for the assessment of individual US signs.

US Signs	ĸ Coefficient Values		
	Min.	Max.	Mean
I-lines	0.26	1.00	0.60
Z-lines	0.05	0.53	0.34
single B-lines	0.37	0.89	0.61
numerous B-lines	0.84	1.00	0.95
Am-lines	0.63	1.00	0.84
pleural line abnormalities	0.26	0.84	0.57
small consolidations	0.68	1.00	0.84
major consolidations	0.79	1.00	0.94
pleural fluid *	0.63	1.00	0.81

* The agreement was assessed for individual lung fields for all US signs except for pleural fluid.

**Table 4:** Publications on the use of LUS in patients with CF.

		Peixoto 2019 [22]	Strzelczuk-Judka 2019 [23]	Peixoto 2020 [24]	Hassanzad 2021 [25]	Ciuca 2022 [26]	Jaworska 2023
**study type**		case study	cross-sectional (observational in 9 cases)	cross-sectional	cross-sectional	cross-sectional	cross-sectional
**number of patients**		2	48	18	30	57	131
**patients’ age**		18 and 21 years	5–18 years	9–22 years	2–>29 years	0.5–18 years	5 weeks –18 years
**patients’ clinical** **condition**		PEx	stable	stable	PEx	stable	stable
**control group**		—	—	—	—	—	+
**interobserver group**		—	—	—	—	—	+
**LUS score**		+	+	+	—	+	+
**correlation between LUS score and**	**CXR**	—	+	—	[+]	—	+
	**chest CT**	(+)	—	+	[+]	+	—
	**PFTs**	(+)	—	+	—	+	+
	**microbiological status**	(+)	—	—	—	—	+

(+) The results of these tests were described in the paper, but due to the insufficient number of cases, neither correlation nor dependence could be calculated. [+] Selected US signs were compared with CXR lesions in relation to chest CT (the scores were not correlated). PEx—pulmonary exacerbation, LUS—lung ultrasound, CXR—chest X-ray, CT—computed tomography, PFTs—pulmonary function tests, + —it was inclued, — —it was not included.

## Data Availability

Data are contained within the article.

## Supplementary Materials

The following supporting information can be downloaded at: https://www.mdpi.com/article/10.3390/jcm12093086/s1, Figure S1: Division of the chest into lung fields; Table S1: Modified Chrispin–Norman score; Table S2: Strength of agreement depending on the value of the ĸ coefficient; Table S3: Guilford’s interpretation of the magnitude of correlation; Figure S2: Total cross-sectional area of consolidations; Figure S3: Cross-sectional area of consolidations in individual lung fields; Figure S4: Distribution of the number of points on the CF LUS score in the study group; Table S4: Number of points on the CF LUS score in individual age groups; Figure S5: Distribution of the number of points on the CF LUS score in individual age groups; Figure S6: Distribution of the number of points on the ultrasound and radiographic scores; Figure S7: Comparison of the number of points in individual age groups for ultrasound and radiographic scores; Table S5: Interobserver agreement for assessing the incidence of I-lines; Table S6: Interobserver agreement for assessing the incidence of Z-lines; Table S7: Interobserver agreement for assessing the incidence of single B-lines; Table S8: Interobserver agreement for assessing the incidence of numerous B-lines; Table S9: Interobserver agreement for assessing the incidence of Am-lines; Table S10: Interobserver agreement for assessing the incidence of pleural line abnormalities; Table S11: Interobserver agreement for assessing the incidence of small consolidations; Table S12: Interobserver agreement for assessing the incidence of major consolidations; Figure S8: Comparison of the total cross-sectional area of consolidations obtained by two sonographers; Table S13: Comparison of ultrasound scores in CF.
