# Supplementary material for: Lung Ultrasound in the Evaluation of Lung Disease Severity in Children with Clinically Stable Cystic Fibrosis: A Prospective Cross-Sectional Study
Source: J Clin Med. 2023 Apr 24;12(9):3086. doi: 10.3390/jcm12093086 (PMC10179222; doi:10.3390/jcm12093086)
Supplement: Supplementary file 1 [file jcm-12-03086-s001.zip › jcm-2193021-supplementary.pdf]

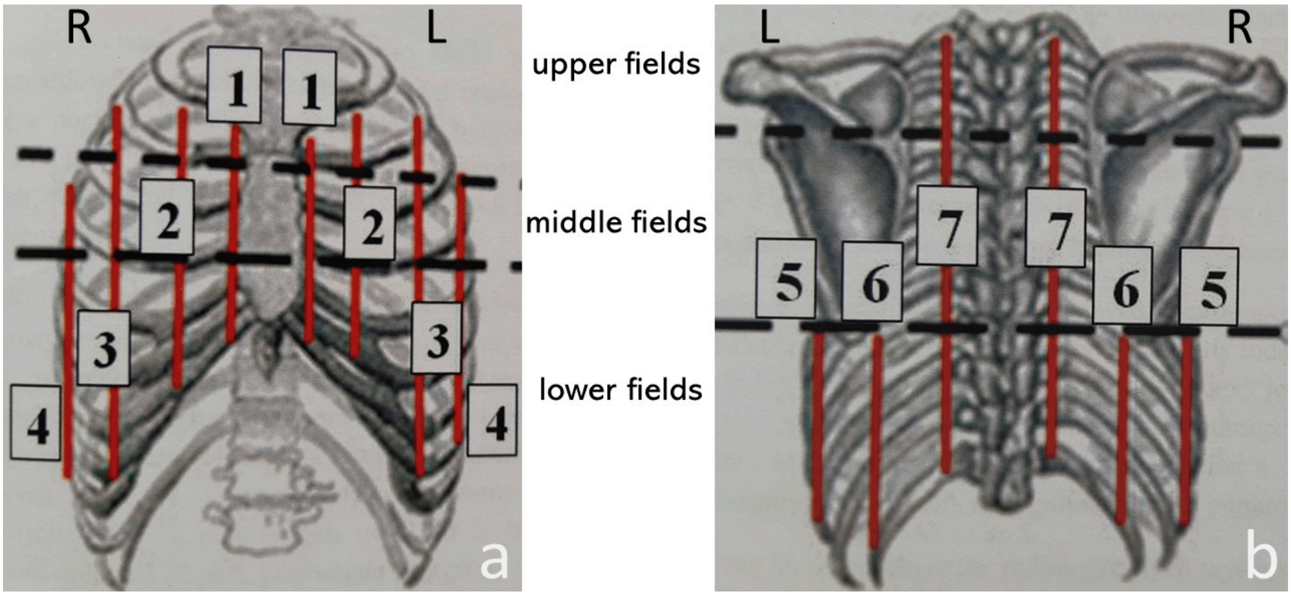

Figure S1 Division of the chest into lung fields [30]

(a) anterior:

- *upper field* – the supraclavicular fossa and the 1st intercostal space (**aUL** – anterior upper left, **aUR** – anterior upper right);
- *middle field* – the 2nd and the 3rd intercostal space (**aML** – anterior middle left, **aMR** – anterior middle right);
- *lower field* – from the 4th intercostal space to the lung base (**aLL** – anterior lower left, **aLR** – anterior lower right)

(b) posterior:

- *upper field* – from the apex of the lung to the 3rd intercostal space (**pUL** – posterior upper left, **pUR** – posterior upper right);
- *middle field* – from the 4th to the 6th intercostal space (**pML** – posterior middle left, **pMR** – posterior middle right);
- *lower field* – from the 7th intercostal space to the lung base (**pLL** – posterior lower left, **pLR** – posterior lower right)

R – right, **L** – left, **1** – parasternal line, **2** – midclavicular line, **3** – anterior axillary line, **4** – midaxillary line,
5 – posterior axillary line, **6** – scapular line, **7** – paravertebral line

Table S1 Modified Chrispin-Norman score [35,36]

feature	not present	present, not marked	marked
**overinflation**			
diaphragmatic depression	0	1	2
chest wall shape	0	1	2
lung fields	0	1	2
**bronchial line shadows**			
right upper zone (RU)	0	1	2
left upper zone (LU)	0	1	2
right lower zone (RL)	0	1	2
left lower zone (LL)	0	1	2
**ring shadows**			
RU	0	1	2
LU	0	1	2
RL	0	1	2
LL	0	1	2
**mottled shadows**			
RU	0	1	2
LU	0	1	2
RL	0	1	2
LL	0	1	2
**large soft shadows**			
RU	0	1	2
LU	0	1	2
RL	0	1	2
LL	0	1	2

maximum score – 38 points

Table S2 Strength of agreement depending on the value of the κ coefficient [45]

κ coefficient value	strength of agreement
< 0.20	slight
0.21 - 0.40	fair
0.41 - 0.60	moderate
0.61 - 0.80	good
0.81 - 1.00	very good

Table S3 Guilford's interpretation of the magnitude of correlation [48]

R-value	interpretation
$\|R\| = 0$	lack of correlation
$0.0 < \|R\| \leq 0.1$	slight, almost negligible correlation
$0.1 < \|R\| \leq 0.3$	low correlation
$0.3 < \|R\| \leq 0.5$	moderate correlation
$0.5 < \|R\| \leq 0.7$	high correlation
$0.7 < \|R\| \leq 0.9$	very high correlation
$0.9 < \|R\| < 1.0$	almost complete correlation
$\|R\| = 1$	complete correlation

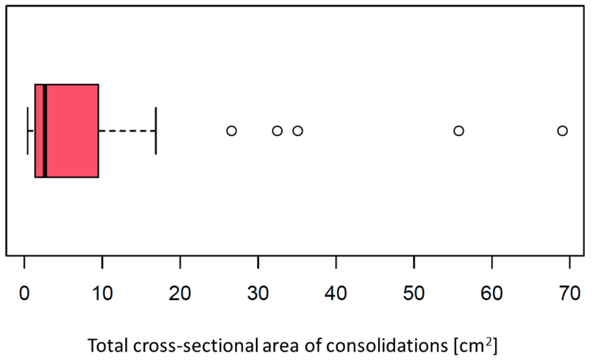

Figure S2. Total cross-sectional area of consolidations

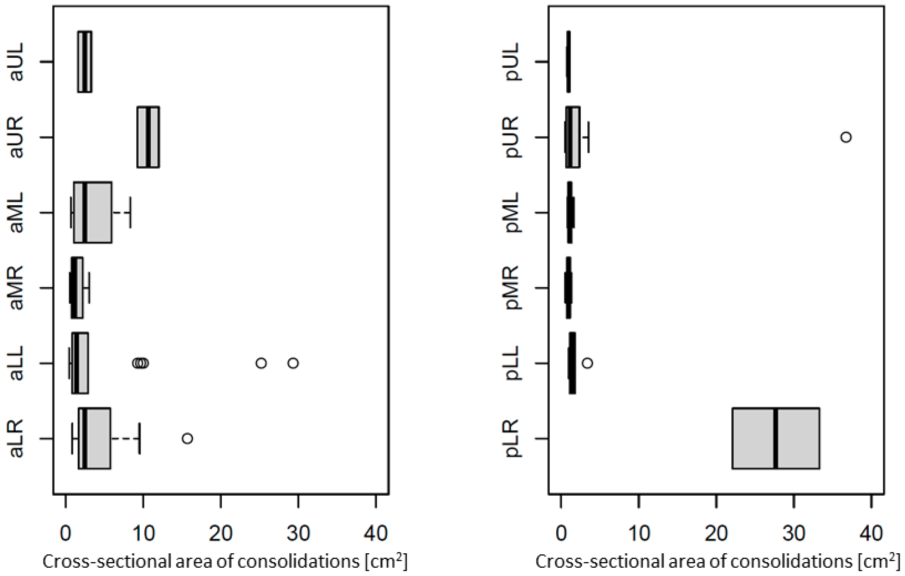

Figure S3. Cross-sectional area of consolidations in individual lung fields

lung fields: **aUL** – anterior upper left, **aUR** – anterior upper right, **aML** – anterior middle left, **aMR** – anterior middle right, **aLL** – anterior lower left, **aLR** – anterior lower right, **pUL** – posterior upper left, **pUR** – posterior upper right, **pML** – posterior middle left, **pMR** – posterior middle right, **pLL** – posterior lower left, **pLR** – posterior lower right

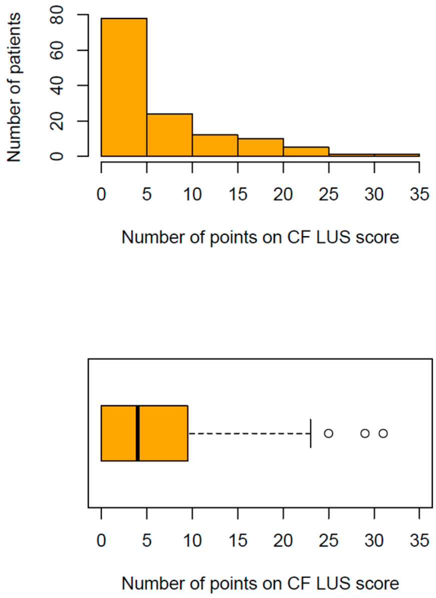

Figure S4. Distribution of the number of points on the CF LUS score in the study group

Table S4. Number of points on the CF LUS score in individual age groups

age group	number of children	number of points on CF LUS score		
		mean ± SD	median [Q1; Q3]	[min; max]
infants (2–12 months)	16	0.13 ± 0.34	0 [0.0; 0.0]	[0; 1]
toddlers (13–36 months)	15	1.13 ± 2.67	0 [0.0; 0.5]	[0; 9]
preschoolers (4–6 years)	29	2.72 ± 3.81	2 [0.0; 3.0]	[0; 18]
middle childhood (7–10 years)	22	6.14 ± 6.36	4 [3.0; 8.8]	[0; 29]
teenagers (10+ years)	49	11.76 ± 6.82	11 [7.0; 16.0]	[0; 31]

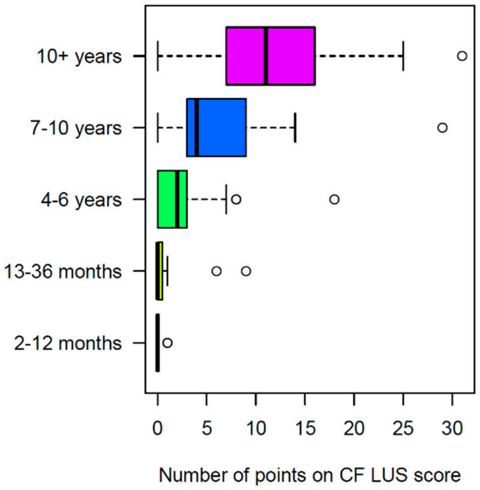

Figure S5. Distribution of the number of points on the CF LUS score in individual age groups

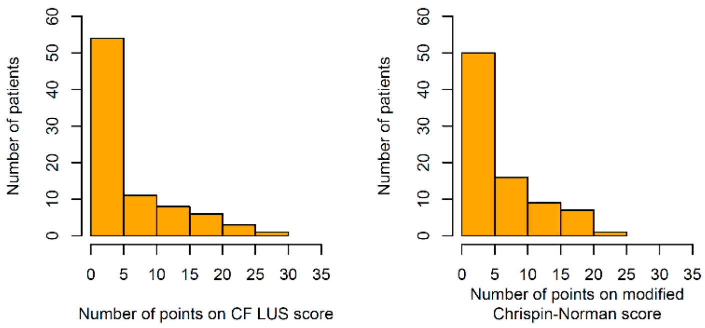

Figure S6. Distribution of the number of points on the ultrasound and radiographic scores

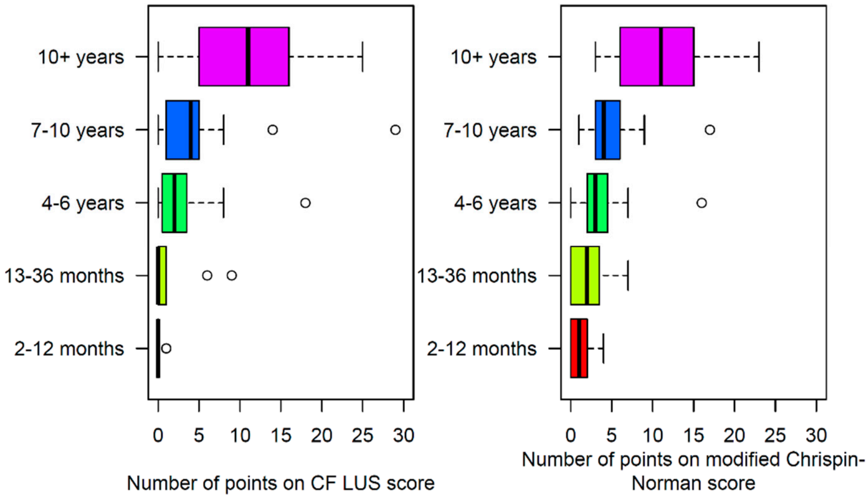

Figure S7. Comparison of the number of points in individual age groups for ultrasound and radiographic scores

Table S5. Interobserver agreement for assessing the incidence of I-lines

lung field	κ values	95% CI	lung field	κ values	95% CI
aUL	0.42	[0.08; 0.69]	aUR	0.53	[0.20; 0.77]
aML	0.63	[0.31; 0.85]	aMR	0.63	[0.31; 0.85]
aLL	0.79	[0.50; 0.94]	aLR	1.00	[0.81; 1.00]
pUL	0.53	[0.20; 0.77]	pUR	0.57	[0.25; 0.81]
pML	0.37	[0.03; 0.65]	pMR	0.26	[-0.08; 0.56]
pLL	0.79	[0.40; 0.94]	pLR	0.68	[0.37; 0.88]
LL-A	0.84	[0.57; 0.97]	RL-A	0.95	[0.72; 1.00]
LL-P	0.84	[0.57; 0.97]	RL-P	0.79	[0.50; 0.94]

κ- kappa coefficient, 95% CI – 95% confidence interval

lung fields: **aUL** – anterior upper left, **aUR** – anterior upper right, **aML** – anterior middle left, **aMR** – anterior middle right, **aLL** – anterior lower left, **aLR** – anterior lower right, **pUL** – posterior upper left, **pUR** – posterior upper right, **pML** – posterior middle left, **pMR** – posterior middle right, **pLL** – posterior lower left, **pLR** – posterior lower right, **LL-A** – left lung – anterior fields, **RL-A** – right lung – anterior fields, **LL-P** – left lung – posterior fields, **RL-P** – right lung – posterior fields

Table S6. Interobserver agreement for assessing the incidence of Z-lines

lung field	κ values	95% CI	lung field	κ values	95% CI
aUL	0.26	[-0.08; 0.56]	aUR	0.16	[-0.18; 0.47]
aML	0.05	[-0.28; 0.38]	aMR	0.53	[0.20; 0.77]
aLL	0.32	[-0.03; 0.61]	aLR	0.47	[0.14; 0.73]
pUL	0.37	[0.03; 0.65]	pUR	0.53	[0.20; 0.77]
pML	0.26	[-0.08; 0.56]	pMR	0.26	[-0.08; 0.56]
pLL	0.37	[0.03; 0.65]	pLR	0.47	[0.14; 0.73]
LL-A	0.53	[0.19; 0.77]	RL-A	0.53	[0.19; 0.77]
LL-P	0.21	[-0.13; 0.52]	RL-P	0.63	[0.31; 0.85]

Table S7. Interobserver agreement for assessing the incidence of single B-lines

lung field	κ values	95% CI	lung field	κ values	95% CI
aUL	0.53	[0.23; 0.84]	aUR	0.53	[0.20; 0.77]
aML	0.63	[0.31; 0.85]	aMR	0.79	[0.50; 0.94]
aLL	0.89	[0.65; 0.99]	aLR	0.68	[0.37; 0.88]
pUL	0.37	[0.03; 0.65]	pUR	0.37	[0.03; 0.65]
pML	0.47	[0.14; 0.73]	pMR	0.53	[0.30; 0.77]
pLL	0.68	[0.37; 0.88]	pLR	0.79	[0.50; 0.94]
LL-A	0.95	[0.72; 1.00]	RL-A	0.95	[0.72; 1.00]
LL-P	0.95	[0.72; 1.00]	RL-P	1.00	[0.81; 1.00]

Table S8. Interobserver agreement for assessing the incidence of numerous B-lines

lung field	κ values	95% CI	lung field	κ values	95% CI
aUL	1.00	[0.82; 1.00]	aUR	1.00	[0.82; 1.00]
aML	0.95	[0.72; 1.00]	aMR	1.00	[0.82; 1.00]
aLL	0.95	[0.72; 1.00]	aLR	0.95	[0.72; 1.00]
pUL	0.95	[0.72; 1.00]	pUR	0.95	[0.72; 1.00]
pML	0.95	[0.72; 1.00]	pMR	0.84	[0.57; 0.97]
pLL	0.89	[0.65; 0.99]	pLR	0.95	[0.72; 1.00]
LL-A	0.95	[0.72; 1.00]	RL-A	0.95	[0.72; 1.00]
LL-P	0.79	[0.50; 0.94]	RL-P	0.79	[0.50; 0.94]

Table S9. Interobserver agreement for assessing the incidence of Am-lines

lung field	κ values	95% CI	lung field	κ values	95% CI
aUL	0.74	[0.44; 0.91]	aUR	0.79	[0.50; 0.94]
aML	0.79	[0.50; 0.94]	aMR	0.79	[0.50; 0.94]
aLL	0.95	[0.72; 1.00]	aLR	0.63	[0.31; 0.85]
pUL	0.89	[0.65; 0.99]	pUR	0.95	[0.72; 1.00]
pML	1.00	[0.81; 1.00]	pMR	0.79	[0.50; 0.94]
pLL	0.79	[0.50; 0.94]	pLR	0.95	[0.72; 1.00]
LL-A	0.84	[0.57; 0.97]	RL-A	0.79	[0.50; 0.94]
LL-P	0.79	[0.50; 0.94]	RL-P	0.84	[0.57; 0.97]

Table S10. Interobserver agreement for assessing the incidence of pleural line abnormalities

lung field	κ values	95% CI	lung field	κ values	95% CI
aUL	0.84	[0.57; 0.97]	aUR	0.79	[0.50; 0.94]
aML	0.58	[0.25; 0.81]	aMR	0.32	[0.00; 0.61]
aLL	0.84	[0.57; 0.97]	aLR	0.63	[0.31; 0.85]
pUL	0.68	[0.37; 0.88]	pUR	0.63	[0.31; 0.85]
pML	0.32	[0.00; 0.61]	pMR	0.37	[0.03; 0.65]
pLL	0.58	[0.25; 0.81]	pLR	0.26	[-0.08; 0.56]
LL-A	0.68	[0.37; 0.88]	RL-A	0.79	[0.50; 0.94]
LL-P	0.53	[0.20; 0.77]	RL-P	0.42	[0.08; 0.69]

Table S11. Interobserver agreement for assessing the incidence of small consolidations

lung field	κ values	95% CI	lung field	κ values	95% CI
aUL	0.89	[0.65; 0.99]	aUR	1.00	[0.81; 1.00]
aML	0.84	[0.57; 0.97]	aMR	0.84	[0.57; 0.97]
aLL	0.74	[0.44; 0.91]	aLR	0.68	[0.37; 0.88]
pUL	0.84	[0.57; 0.97]	pUR	0.95	[0.72; 1.00]
pML	0.79	[0.50; 0.94]	pMR	0.84	[0.57; 0.97]
pLL	0.79	[0.50; 0.94]	pLR	0.89	[0.65; 0.99]
LL-A	0.75	[0.50; 0.91]	RL-A	0.84	[0.57; 0.97]
LL-P	0.47	[0.14; 0.73]	RL-P	0.68	[0.37; 0.88]

Table S12. Interobserver agreement for assessing the incidence of major consolidations

lung field	κ values	95% CI	lung field	κ values	95% CI
aUL	1.00	[0.81; 1.00]	aUR	1.00	[0.81; 1.00]
aML	0.95	[0.72; 1.00]	aMR	0.89	[0.65; 0.99]
aLL	0.95	[0.72; 1.00]	aLR	0.79	[0.50; 0.94]
pUL	0.95	[0.72; 1.00]	pUR	1.00	[0.81; 1.00]
pML	0.95	[0.72; 1.00]	pMR	0.95	[0.72; 1.00]
pLL	0.89	[0.65; 0.99]	pLR	0.95	[0.72; 1.00]
LL-A	1.00	[0.81; 1.00]	RL-A	1.00	[0.81; 1.00]
LL-P	1.00	[0.81; 1.00]	RL-P	1.00	[0.81; 1.00]

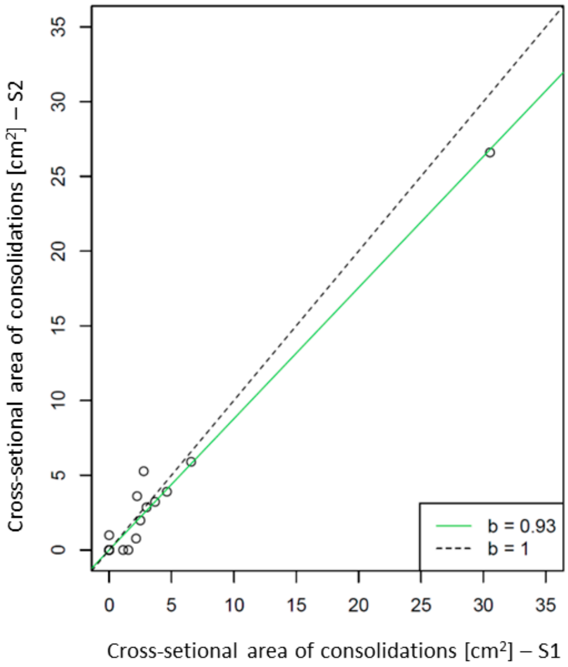

Figure S8. Comparison of the total cross-sectional area of consolidations obtained by two sonographers

S1 – first sonographer, S2 – second sonographer, b – slope of simple linear regression, 95%CI [0.81; 1.05]

Table S13 Comparison of ultrasound scores in CF

		Peixoto 2019 [22]	Strzelczuk-Judka 2019 [23]	Peixoto 2020 [24]	Ciuca 2022 [26]	Jaworska 2023
**assessed ultrasound signs**	**numerous B-lines**	+	+	+	+	+
	**confluent B-lines**	—	+	—	+	+
	**consolidations**	+	+	+	+	+
	**atelectasis distinguished from other consolidations**	—	—	—	+	—
	**equivalent of bronchiectasis**	—	—	—	*	Am-lines
	**pleural line abnormalities**	—	+	+	—	+
	**pleural fluid**	—	+	—	—	—
**number of assessed lung fields**		12	4	12	12	12
**max. possible score**		36	40	36	—	44
**max. number of points obtained in the study group**		18	16	18	21	31
**correlation between LUS score and**	**CXR score**	—	R = 0.52	—	—	R = 0.87
	**chest CT score**	—	—	par. cor. = 0.61	R = 0.87	—
	**age**	—	—	—	—	R = 0.70
	**LCI**	—	—	—	R = 0.80	R = 0.59
	**FEV1**	—	—	par. cor. = -0.54	R = -0.65	R = -0.63

* The following US signs were considered the equivalent of bronchiectasis: 1. > 3 B-lines and 1 coalescent B-line; 2. > 2 coalescent B-lines; 3. bronchial wall thickening or subpleural consolidations < 10 mm

LUS – lung ultrasound, CXR – chest X ray, CT – computed tomography, LCI – lung clearance index, FEV1 – forced expiratory volume in 1 second, R – Pearson's correlation coefficient, par. cor. – partial correlation
